# Chemically active wetting

**DOI:** 10.1073/pnas.2403083122

**Published:** 2025-04-09

**Authors:** Susanne Liese, Xueping Zhao, Christoph A. Weber, Frank Jülicher

**Affiliations:** ^a^Faculty of Mathematics, Natural Sciences, and Materials Engineering, and Institute of Physics, University of Augsburg, Augsburg 86159, Germany; ^b^Department of Mathematical Sciences, University of Nottingham, Ningbo 315100, China; ^c^Max Planck Institute for the Physics of Complex Systems, Dresden 01187, Germany; ^d^Center for Systems Biology Dresden, Dresden 01307, Germany; ^e^Cluster of Excellence Physics of Life, Technical University Dresden, Dresden 01062, Germany

**Keywords:** wetting, nonequilibrium thermodynamics, phase separation

## Abstract

Wetting of liquid droplets on passive surfaces is ubiquitous in our daily lives, and the governing physical laws are well understood. When surfaces become active, however, the governing laws of wetting remain elusive. Here, we propose chemically active wetting as a class of active systems where the surface is active due to a binding process that is maintained away from equilibrium. We derive the corresponding nonequilibrium thermodynamic theory and show that active binding fundamentally changes the wetting behavior, leading to steady, nonequilibrium states with droplet shapes reminiscent of a pancake or a mushroom. The origin of such anomalous shapes can be explained by mapping to electrostatics, where pairs of binding sinks and sources correspond to electrostatic dipoles along the triple line. This is an example of a more general analogy, where localized chemical activity gives rise to a multipole field of the chemical potential. The underlying physics is relevant for cells, where droplet-forming proteins can bind to membranes accompanied by the turnover of biological fuels.

## Significance

Wetting is ubiquitous in both the macroscopic and microscopic world. Wetting behavior that significantly deviates from the well-studied properties of passive systems arises when maintaining a system away from equilibrium. Here, we study active wetting, where active binding processes between droplet and surface maintain the system away from equilibrium. Our work demonstrates the qualitative differences between active and passive wetting phenomena. We reveal that wetting on active surfaces results in complex droplet shapes that deviate from the spherical cap shape seen in passive droplets.

From water droplets spreading on glass surfaces to raindrops rolling off plant leaves, wetting phenomena are ubiquitous in our daily lives. On macroscopics scales, the laws of wetting on passive surfaces are well understood. The shape of a wetted droplet follows a spherical cap and the contact angle between the cap and the surface is governed by the law of Young–Dupré relating the surface tensions at the triple line (1–4). The stationary shape of a wetted drop can however deviate from a spherical cap in the presence of gravitation (5), visco-plasticity (6) and heterogeneous or patterned surfaces (7).

Wetting phenomena are not limited to solid surfaces in the macroscopic world; they also manifest at mesoscopic scales on biological surfaces such as membranes, where micrometer-sized coacervate droplets wet lipid bilayer surfaces. Wetting interactions on such scales can even deform membrane vesicles (8–10), give rise to a large variety of complex droplet and vesicle shapes (11) and modulate lipid packing in the membrane (12). In cells, wetting of biomolecular condensates occurs on membrane surfaces of organelles (13–15) and the cell’s membrane (11, 16–19). A key property of membranes is that molecules, in particular droplet components, can bind to specific receptors embedded in the membrane. Theoretical studies showed that membrane binding can give rise to surface phase transitions and an altered wetting dynamics (17, 20). In cells, surface binding often involves chemical activity that maintains binding away from equilibrium, suggesting more complex phenomena in active wetting. This activity is typically supplied by biological fuels such as ATP or GTP (21–23).

Active biophysical systems exhibit a rich set of phenomena (24–27). Active drops can divide (28–30), form liquid shells (31–33), suppress coarsening (31, 34, 35), and regulate wetting on surfaces (36, 37). In phase-separated systems where chemical reactions are maintained away from equilibrium, the mismatch of chemical and phase equilibrium leads to spatial fluxes of the components, even in steady state (38). How fluxes that are driven by active binding processes located at the interface between a droplet and a surface affect wetting remains elusive.

To understand the interplay between active binding and membrane wetting, we introduce a class of active systems, where droplets undergo chemically active wetting, and derive the corresponding nonequilibrium thermodynamic theory. We draw a formal analogy to electrostatics, suggesting that the triple line acts as a source multipole, generating a spatial pattern of chemical potential. The resulting diffusion fluxes deform a spherical cap-shaped droplet at equilibrium to shapes reminiscent of a pancake or a mushroom at nonequilibrium steady state.

## Theory of Active Wetting

**Fig. 1.**
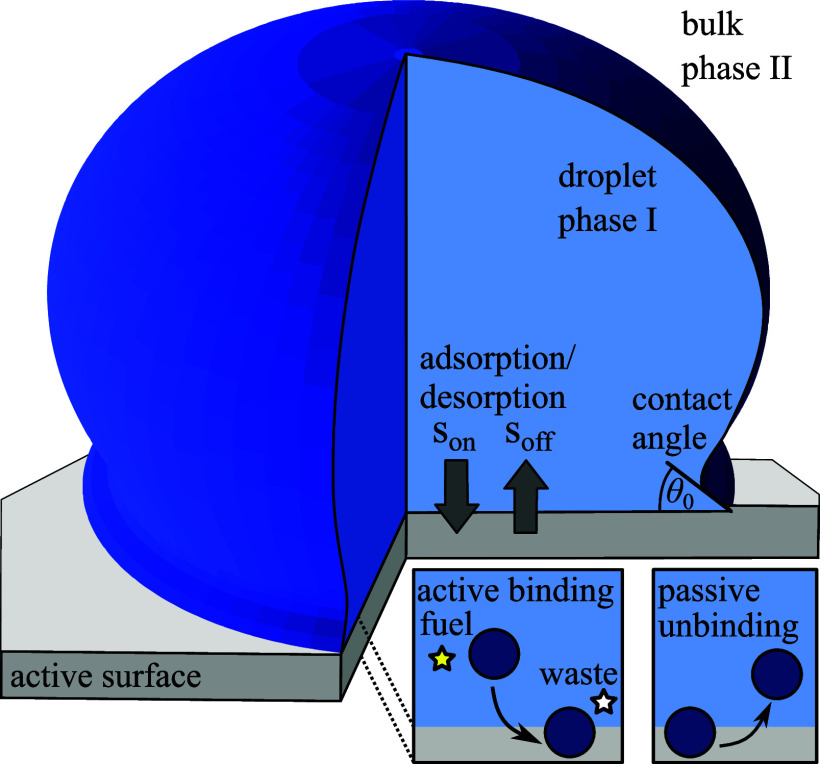
Schematic illustration of wetting on chemically active surfaces. The droplet can wet the planar surface containing a droplet (dense phase I) surrounded by a dilute phase (II). The droplet components bind to the membrane, forming a two-dimensional layer at the interface between bulk and membrane. The unbinding flux $s_{\mathrm{off}}$ is passive, while the binding flux $s_{\mathrm{on}}$ is governed by active processes, which can be realized by the consumption/production of fuel/waste.

We consider a binary solute-solvent mixture that can phase-separate in the bulk and which is in contact with a membrane surface (Fig. 1). In a finite system, a droplet-phase rich in solutes can coexist with a dilute phase in the bulk and wet the surface, exhibiting a local contact angle *θ*_0_. Moreover, the solutes are able to bind to and detach from the membrane at a rate described by the net desorption flux *s*. This binding process can be passive, settling at binding equilibrium, or it can be active.

The dynamics of active wetting can be described by a continuum theory where the fields of volume fraction in the bulk *ϕ* and the area fraction on the membrane $\phi_{m}$ are determined by the conservation laws:

[1a] $$\begin{array}{cc} \partial_{t}\phi & =-\nabla \cdot j\;, \end{array}$$

[1b] $$\begin{array}{cc} \partial_{t}\phi_{m} & =-\nabla_{\|}\cdot j_{m}-s\;, \end{array}$$

where j and $j_{m}$ are the diffusive fluxes in bulk and membrane, respectively. The gradient vector in the membrane plane is denoted by $\nabla_{\|}$.

The coupling energy $\Omega (\phi_{0})$ between bulk and the membrane surface, located at *z* = 0, sets a boundary condition for the bulk volume fraction *ϕ* at the surface

[1c] $$\begin{array}{c} n\cdot \nabla \phi {|}_{z=0}=\frac{1}{\kappa }\frac{\partial \Omega }{\partial \phi_{0}}, \end{array}$$

with $\phi_{0}=\phi \left(z=0\right)$, *κ* characterizing the free energy cost for spatial inhomogeneities in *ϕ* and $n={\left(0,0,1\right)}^{T}$ the normal vector of the membrane. We note that the coupling energy is related to the local contact angle *θ*_0_; more details are given in *Materials and Methods*.

For solutes being conserved in membrane and bulk, the net desorption flux *s* is related to the normal component of the diffusive bulk flux at the membrane surface:

[1d] $$\begin{array}{c} n\cdot j=\frac{\nu }{\nu_{m}}s\;, \end{array}$$

with *ν* and $\nu_{m}$ are the molecular volume and molecular area, respectively.

The net desorption flux is composed of the difference between an unbinding and a binding flux, $s=s_{\mathrm{off}}-s_{\mathrm{on}}$. In passive systems, the two fluxes are linked by the detailed-balance of the rates, which ensures *s* = 0 at chemical equilibrium, corresponding to $\mu_{m}=\mu$, with the chemical potentials in bulk, *μ*, and membrane, $\mu_{m}$. For an active system detailed balance of the rates is broken

[1e] $$\begin{array}{c} \frac{s_{\mathrm{on}}}{s_{\mathrm{off}}}\neq \;\exp\left[-\frac{\mu_{m}-\mu }{k_{B}T}\right]\;. \end{array}$$

Thus *s* ≠ 0 for $\mu \neq \mu_{m}$ such that binding is driven away from equilibrium. Here, $k_{B}$ denotes the Boltzmann constant and *T* the temperature.

The diffusive fluxes in bulk and membrane

[1f] $$\begin{array}{cc} j & =-\Lambda (\phi )\nabla \mu \;, \end{array}$$

[1g] $$\begin{array}{cc} j_{m} & =-\Lambda_{m}\left(\phi_{m}\right)\nabla_{\|}\mu_{m}\;, \end{array}$$

are driven by gradients in the bulk and membrane chemical potential *μ* and $\mu_{m}$, respectively, where Λ and $\Lambda_{m}$ denote respective kinetic coefficients. Their volume and area fraction dependence are given in *Materials and Methods*.

**Fig. 2.**
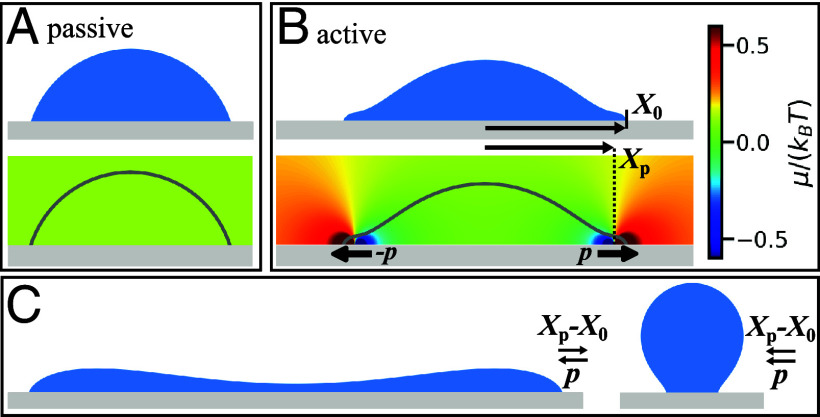
Wetting on passive and chemically active surfaces. Figure panels are obtained from solving the shape equation (Eq. **5**) for a two-dimensional system. Details of the numerical method are given in *SI Appendix*, section II. (*A*) Equilibrium droplet in a passive system with a contact angle $\theta_{0}=0.4\pi$. The corresponding chemical potential is homogenous in space. (*B*) Stationary droplet on an active surface with $p=5\overset{¯}{p}$, $X_{p}-X_{0}=-1\ell$. The chemical potential is shown as a color map. (*C*) Droplet on an active surface, with $p=-5\overset{¯}{p}$, $X_{p}-X_{0}=1\ell$ and $p=-5\overset{¯}{p}$, $X_{p}-X_{0}=-0.2\ell$, with $\overset{¯}{p}=\Lambda \nu_{m}k_{B}T\ell /\nu$.

For a passive surface without any active binding processes, the steady-state solution of (Eq. **1**) corresponds to thermodynamic equilibrium. It is characterized by a homogeneous chemical potential that is identical between bulk and membrane implying that the diffusive fluxes j and $j_{m}$, and the net desorption flux *s* are each zero. In this case, the wetted droplet takes the shape of a spherical cap and the contact angle *θ*_0_ fulfills the law of Young–Dupré (Fig. 2*A*).

By keeping the binding away from chemical equilibrium, the fluxes j, $j_{m}$ and *s* remain nonzero in steady state and we observe a qualitative change of the wetting behavior. The droplet shape can now differ significantly from a spherical cap, caused by a position-dependent chemical potential.

## Mapping to Electrostatics

**Fig. 3.**
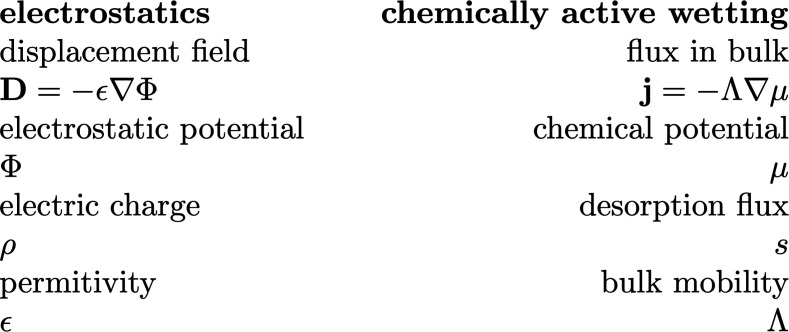
Mapping to electrostatics. Correspondence of quantities from electrostatics (*Left*) and wetting at active surfaces at steady state (*Right*).

The shape of wetting droplets on an active surface can be understood by drawing an analogy to electrostatics. To this end, we consider a charge-free, linear dielectric medium adjacent to a nonconducting, nonpolarizable medium. The interface is heterogeneously charged with a charge area density ρ(x,y). According to Gauss’s law, the displacement field D fulfills ∇·D=0 in the absence of free charges and n·D=ρ at the interface (for more details, see *SI Appendix*, section IV). Comparing the electrostatic equations with the dynamic equations for active wetting Eq. **1** at steady state ($\partial_{t}\phi =0$, $\partial_{t}\phi_{m}=0$) suggests a mapping between electrostatics and active wetting, which is depicted in Fig. 3. Specifically, the net desorption flux *s* generates a position-dependent chemical potential *μ* in the same way as a charge density *ρ* gives rise to an electrostatic potential Φ. Therefore, the far field of the chemical potential corresponds to the electrostatic potential field of a multipole.

To illustrate the mapping to electrostatics further, we consider a two-dimension system for simplicity. In this case, a two-dimensional droplet interacts with a one-dimensional membrane or equivalently, a two-dimensional electrostatic potential resulting from a one-dimensional line charge density. The three-dimensional case is discussed in *SI Appendix*, section V.

The multipole generated by the net desorption flux gives rise to a chemical potential profile which is governed to leading order by two dipoles positioned at $\pm X_{\text{p}}$. For constant mobility Λ, this chemical potential can be written as (*SI Appendix*, section V.A):

[2] $$\begin{array}{cc} \mu (x,z)= & \frac{p\;\nu }{2\pi \nu_{m}\Lambda }[\frac{x-X_{p}}{{\left(x-X_{p}\right)}^{2}+z^{2}}\\ & -\frac{x+X_{p}}{{\left(x+X_{p}\right)}^{2}+z^{2}}]+\mu_{c}\;. \end{array}$$

This chemical potential profile corresponds to the superposition of two dipole moments with opposite orientation and magnitude *p*. The two dipoles have a distance $2X_{p}$ from each other. In Eq. **2**, *x* and *z* denote the lateral and horizontal coordinates and $\mu_{c}$ is a constant offset, that acts as a Lagrange multiplier to ensure a symmetric droplet shape.

The chemical potential profile Eq. **2** can be derived from the multipole moments of the net desorption flux *s*. The monopole $q=\int_{-\infty }^{\infty }dx\;s\left(x\right)$ has to vanish due to particle conservation in a stationary system. The dipole moment of the whole active surface also vanishes due to the mirror symmetry of the droplet, which is formally written as $\int_{-\infty }^{\infty }dx\;xs\left(x\right)=0$. Thus, the first nonvanishing moment is the quadrupole moment. The quadrupole moment is generated by two oppositely oriented dipoles of equal magnitude

[3] $$\begin{array}{c} p=\int_{0}^{\infty }dx\;xs\left(x\right)\;, \end{array}$$

that are placed at $x=\pm X_{p}$, with the dipole position given as

[4] $$\begin{array}{c} X_{p}=\frac{\int_{0}^{\infty }dx\;x^{2}\;s\left(x\right)}{2\int_{0}^{\infty }dx\;xs\left(x\right)}\;. \end{array}$$

Using the magnitude of the dipole moments and their positions, we obtain the potential profile given in Eq. **2**. We note that the position $X_{p}$ of the dipole and the position of the droplet interface *X*_0_ are in general not identical, due to the potential asymmetry of the net desorption flux *s* around the triple point. We furthermore note, that *p* and $X_{p}$ are not independent as they are both determined by the flux *s*. In turn, any combination of *p* and $X_{p}$ can be obtained by choosing the appropriate functional form of s(x).

## Droplet Shapes on Active Surfaces

We first discuss an effective droplet model describing the interface shape. The droplet shape is determined by the position-dependent chemical potential that results from the active binding processes with the surface. Note that there are no chemical reactions in the bulk. Therefore, we can consider the droplet interface between the dense droplet phase and the dilute phase to be at local equilibrium, implying a Gibbs–Thomson relation (39). The mean interface curvature *H* at a point on the droplet surface is related to the local chemical potential as $\left(\mu -\mu_{0}\right)/k_{B}T=\ell H$, with *μ*_0_ the equilibrium chemical potential in the thermodynamic limit and where the capillary length $\ell =\nu \gamma_{0}/\left[k_{B}T\left(\phi^{\text{I}}-\phi^{\text{II}}\right)\right]$, is set by the ratio of the surface tension of the planar interface *γ*_0_ and the thermal energy. Using an arc length parameterization of the droplet shape (x(S),z(S)), with the arc length *S*, the mean curvature H=−dθ/dS, and *θ* as the angle to the horizontal *x*-axis, we find the following shape equations:

[5a] $$\begin{array}{cc} \frac{\mathrm{dx}}{\mathrm{dS}} & =\;\cos\theta \;, \end{array}$$

[5b] $$\begin{array}{cc} \frac{\mathrm{dz}}{\mathrm{dS}} & =\;\sin\theta \;, \end{array}$$

[5c] $$\begin{array}{cc} \frac{d\theta }{\mathrm{dS}} & =-\frac{1}{\ell }\frac{\mu \left(x,z\right)-\mu_{0}}{k_{B}T}\;, \end{array}$$

with the boundary conditions

[5d] $$\begin{array}{cc} & x\left(0\right)=-X_{0}\;,\;z\left(0\right)=0\;,\;\theta \left(0\right)=\theta_{0}\;, \end{array}$$

[5e] $$\begin{array}{cc} & \theta (S_{\mathrm{mid}})=0\;, \end{array}$$

with $S_{\mathrm{mid}}$ denoting the mid point of the droplet interface and *X*_0_ is the position of the triple line. For a given *X*_0_, the offset of the chemical potential $\mu_{c}$ in Eq. **2** has to be adjusted to match the boundary condition at $S_{\mathrm{mid}}$. We note that the area, i.e., the two-dimensional volume, can be specified instead of *X*_0_. In this case, *X*_0_ is a free parameter and $\mu_{c}$ acts as a Lagrange multiplier to impose a fixed volume. Note that in the following, we consider for simplicity a symmetric double well free energy density, implying $\mu_{0}=0$. Fig. 2*C*) shows two examples of strongly deformed droplets. Depending on the choice of the parameters *p* and $X_{p}-X_{0}$, a tightly constricted droplet shape or a wide droplet with an inward bulge in the center is created.

## Comparison of the Effective Droplet Model and the Continuum Model

To compare the results of the effective droplet model with numerical simulations of the continuum model, we have to make a specific choice of the binding flux *s*. We introduce an external free energy $\Delta \mu_{\mathrm{act}}$, such that

[6] $$\begin{array}{c} \frac{s_{\mathrm{on}}}{s_{\mathrm{off}}}=\;\exp\left[-\frac{\mu_{m}-\left(\mu +\Delta \mu_{\mathrm{act}}\right)}{k_{B}T}\right]\;. \end{array}$$

For $\Delta \mu_{\mathrm{act}}\neq 0$, Eq. **6** breaks the detailed balance of the rates. It reflects the idea of binding processes that are maintained away from equilibrium by coupling of binding to active processes. This case can be realized for example by a chemical fuel (32, 40, 41), where binding or unbinding turns the fuel to a reaction product. To ensure that $\Delta \mu_{\mathrm{act}}$ is phase-dependent, we choose

[7] $$\begin{array}{c} \Delta \mu_{\mathrm{act}}=\chi_{\mathrm{act}}k_{B}T\phi_{0}, \end{array}$$

where $\chi_{\mathrm{act}}$ denotes the activity parameter and *ϕ*_0_ is the bulk volume fraction at the membrane surface. Note that our choice with $\chi_{\mathrm{act}}>0$ corresponds to a system where the active contribution $\Delta \mu_{\mathrm{act}}$ to the chemical potential is larger inside the dense phase compared to the droplet surrounding. We consider positive and negative $\Delta \mu_{\mathrm{act}}$ which describe the tendency to enrich or deplete the membrane surface by active binding.

**Fig. 4.**
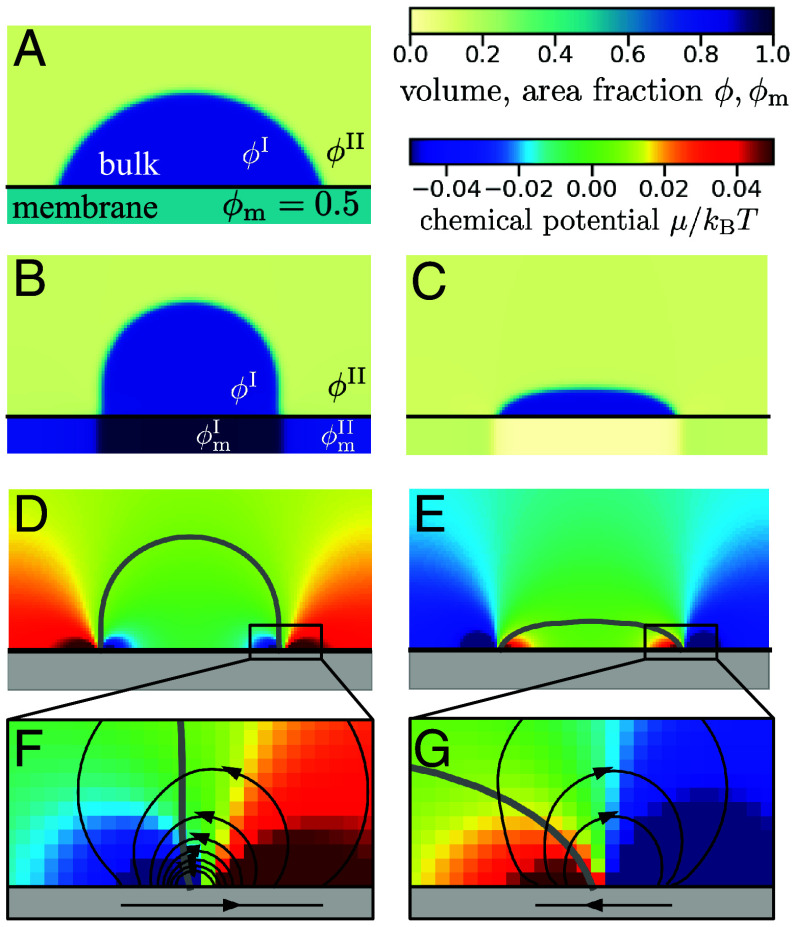
Wetting on passive and chemically active surfaces. All figure panels are obtained from solving Eq. **1** numerically; details of the numerical method are given in *SI Appendix*, section II. (*A*) Equilibrium droplet in a passive system with $\chi_{\mathrm{act}}=0$. (*B* and *C*) Stationary droplets for chemically active binding with $\chi_{\mathrm{act}}=-6$ (*B*) and $\chi_{\mathrm{act}}=4$ (*C*). (*D* and *E*) Chemical potential map that corresponds to the stationary droplets shown in subfigures (*B*) and (*C*). The droplet shape is indicated as a black line. (*F*) and (*G*) Chemical potential map in the vicinity of the contact line. The fluxes in bulk and in the membrane that are caused by gradients of the chemical potential are indicated by black arrows. For better visibility, the membrane is shown extended in height in all subfigures.

When maintaining binding away from chemical equilibrium, ($\Delta \mu_{\text{act}}\neq 0$), we find a nonequilibrium steady state with position-dependent chemical potentials that drive diffusive fluxes in the membrane and the bulk (Fig. 4*B*–*G*). Such fluxes are most pronounced near the triple line. Depending on the value of $\Delta \mu_{\text{act}}>0$, we find shapes that are qualitatively different from a passive system with the contact line expanding or contracting relative to the passive case. For the chemically active surface, we observe droplet shapes that are reminiscent of a pancake or a mushroom, respectively.

**Fig. 5.**
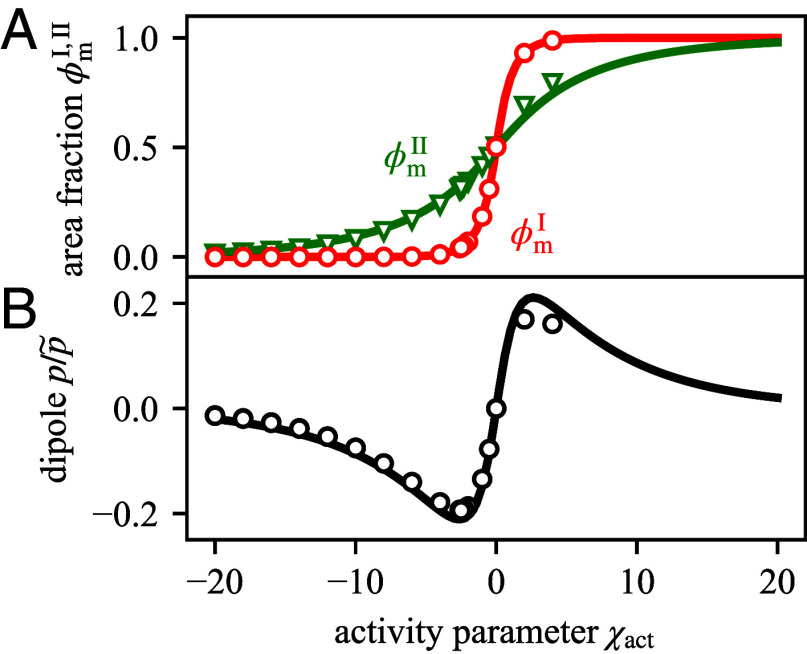
Activity parameter affects the surface volume fractions and magnitudes of sink-source dipole. Results are obtained using the sharp interface model (solid lines) leading to Eqs. **3** and **8**, and continuous simulations Eq. **1** (open symbols). (*A*) The membrane area fractions $\phi_{m}^{\text{I}}$, $\phi_{m}^{\text{II}}$ increases with the activity parameters $\chi_{\text{act}}$ as it promotes binding to the surface. (*B*) The sink-source dipole changes sign at $\chi_{\text{act}}=0$ and vanishes for large $|\chi_{\text{act}}|$ because the active surface gets either depleted or fully occupied in both domains I and II. The dipole is scaled by $\tilde{p}=\lambda_{0}^{2}k_{0}$.

The dipole moment *p* of the active surface is caused by the mismatch of the membrane area fractions $\phi_{m}^{\text{I}}$ and $\phi_{m}^{\text{II}}$ adjacent to the dense and dilute bulk phase. To estimate $\phi_{m}^{\text{I},\;\text{II}}$, we describe the bulk droplet in the limit of a sharp interface leading to the dense and dilute steady state values $\phi^{\text{I}}$ and $\phi^{\text{II}}$. We fix the bulk chemical potential *μ* far from the surface to be constant. Far from the contact line, the system becomes homogeneous even in the active case. The lateral diffusive membrane flux must therefore vanish. Subsequently the net desorption flux vanishes as well, which implies $s_{\mathrm{on}}=s_{\mathrm{off}}$. This results in the following relationship:

[8] $$\begin{array}{c} \mu_{m}-\mu -\chi_{\mathrm{act}}k_{B}T\phi^{\text{I},\;\text{II}}=0\;, \end{array}$$

where $\mu_{m}$ is a function of $\phi_{m}^{\text{I},\;\text{II}}$. The values of $\phi_{m}^{\text{I},\;\text{II}}$ that we determine based on Eq. **8** agree well with the simulation results (Fig. 5*A*). Furthermore, using a sharp interface model, we find an analytic approximation for the dipole moment (see *SI Appendix*, section VI.B for details)

[9] $$\begin{array}{c} p≃\left(D_{m}^{\text{I}}+D_{m}^{\text{II}}\right)\frac{\phi_{m}^{\text{I}}-\phi_{m}^{\text{II}}}{2}\;, \end{array}$$

where $D_{m}^{\text{I},\;\text{II}}$ denote the diffusion constants in a surface of area fraction $\phi_{m}^{\text{I},\;\text{II}}$. Fig. 5*B*) shows that the analytic results obtained from the sharp interface model agree well with the numerical solution of the continuum model (Eq. **1**). We see that the magnitude of the dipole moment *p* exhibits a maximum around $\chi_{\mathrm{act}}=\pm 2.5$. For $\chi_{\mathrm{act}}=0$, the dipole vanishes, resulting in a spatially constant chemical potential, $\mu =\mu_{c}$ (Eq. **2**), in agreement with the key characteristic of passive systems in equilibrium. For large $|\chi_{\mathrm{act}}|$ the dipole vanishes as well, since the surface in both domains I and II gets either depleted ($\phi_{m}\to 0$ for negative $\chi_{\mathrm{act}}$), or fully occupied ($\phi_{m}\to 1$ for positive $\chi_{\mathrm{act}}$). Thus, in both cases, the difference between $\left(\phi_{m}^{\text{I}}-\phi_{m}^{\text{II}}\right)$ becomes small leading to a vanishing magnitude of the dipole moment according to Eq. **9**.

The activity parameter $\chi_{\text{act}}$ also affects the position of the dipole

**Fig. 6.**
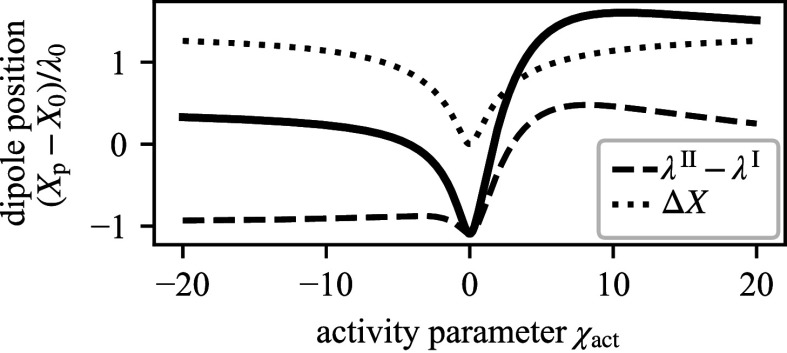
Dipole position depends on activity parameter. Using the sharp interface model, we calculate the difference between the dipole position $X_{p}$ and the droplet interface at the membrane *X*_0_ as a function of the activity parameter; *λ*_0_ is the scale of the reaction–diffusion lengths. The difference $X_{p}-X_{0}$ consists of a symmetric term ΔX and a term that is not symmetric with respect to $\chi_{\mathrm{act}}$, due to the asymmetric contribution of the reaction–diffusion length scales.

[10] $$\begin{array}{c} X_{p}=X_{0}+\Delta X+\lambda^{\text{I}}-\lambda^{\text{II}}\;, \end{array}$$

relative to the triple line at *X*_0_ by a symmetric contribution $\Delta X\left(\chi_{\text{act}}\right)=\Delta X\left(-\chi_{\text{act}}\right)$ and an in general asymmetric contribution from the reaction–diffusion length scales $\lambda^{\text{I,II}}\left(\chi_{\text{act}}\right)$ (Fig. 6; see *SI Appendix*, section VI.B for the expressions of ΔX and $\lambda^{\text{I,II}}$. The dipole can be deflected to the left or the right of the triple line. The asymmetry of this deflection with the activity parameter results from reaction rate coefficients and diffusivities depending on volume and area fractions that vary between the domains I and II (Fig. 6). The changes in the droplet position are accompanied by pronounced changes in droplet shape in the vicinity of the triple line. We find a rather flat, pancake-like drop with a positive local curvature at the triple line when *p* and $\left(X_{p}-X_{0}\right)$ have different signs. Once both have the same sign, the drop has a negative curvature at the triple line leading to mushroom shapes.

To characterize the droplet shape on an active surface, we introduce the contact angle of active wetting $\theta_{\mathrm{act}}$, which obeys

[11] $$\begin{array}{c} \frac{X_{0}^{2}}{A}=\frac{\theta_{\mathrm{act}}-\sin\left(\theta_{\mathrm{act}}\right)\cos\left(\theta_{\mathrm{act}}\right)}{\sin^{2}\left(\theta_{\mathrm{act}}\right)}\;, \end{array}$$

where *A* is the area of the droplet, i.e., the two-dimensional equivalent of the droplet volume. The contact angle $\theta_{\mathrm{act}}$ becomes the local contact angle *θ*_0_ when the droplet wets a passive surface leading to a circular cap shape.

**Fig. 7.**
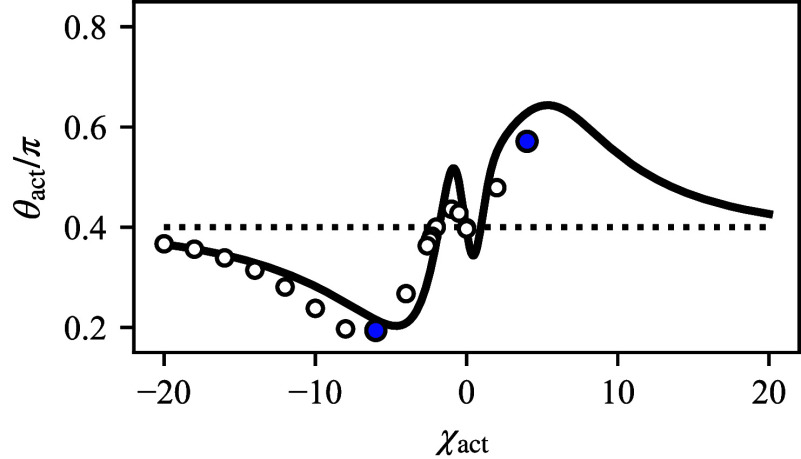
Shape of droplet on active surfaces. The active contact angle depends in a nonmonotonic way on the activity parameter $\chi_{\mathrm{act}}$. For $|\chi_{\mathrm{act}}|\to \infty$, $\theta_{\mathrm{act}}$ approaches the passive value *θ*_0_, as the dipole moment vanishes. Droplet shapes are obtained using the sharp interface model (solid lines) using Eq. **5**, and continuous simulations Eq. **1** (open symbols). The data points shown in blue correspond to the numerical results shown in Fig. 4 *B* and *C*). Results obtained using the sharp interface model are shown for a fixed value $X_{0}/\lambda_{0}=20$. In the numerical simulations, $X_{0}/\lambda_{0}$ varies between 18 and 36. The dashed line indicates the passive contact angle *θ*_0_.

The contact angle $\theta_{\mathrm{act}}$ and thus the droplet shape is controlled by the activity parameter $\chi_{\mathrm{act}}$. For large and negative $\chi_{\mathrm{act}}$, $\theta_{\mathrm{act}}$ is decreased, indicating a pancake shape while for large and positive $\chi_{\mathrm{act}}$, the active contact angle in enhanced corresponding to a mushroom shape (Fig. 7). The results of the sharp interface model (solid line) agree well with the numerical calculations for a continuous interface (open circles).

In summary, our key finding is that the active binding ($\chi_{\mathrm{act}}\neq 0$) leads to deviations of the active angle $\theta_{\text{act}}$ from the local contact angle *θ*_0_. This deviation results from a nonvanishing sink-source dipole at the triple line and an inhomogeneous chemical potential. We note that deviations from the local contact angle by the active process are generic while the precise relation between activity $\chi_{\mathrm{act}}$ and the dipole *p* (Fig. 5), or the nonlinearities of the effective contact angle on $\chi_{\mathrm{act}}$ (Fig. 7) depend on the specific choice of the net desorption flux *s* (Eq. **6**).

## Active Droplet Wetting in Experiments

An open question is how to experimentally realize an active system where wetting and, thereby, the droplet shape can be controlled by active binding processes to a surface. The key ingredient is a chemical substance that can form droplets and adhere to a surface. Molecules can bind and unbind, and both are maintained away from chemical equilibrium. This can be achieved, for example, through the use of a chemical fuel that drives binding and unbinding.

Active wetting may play a role in cell biology. Potential examples are the synaptic vesicle clusters and stress granules in living cells. These systems exhibit the key properties necessary for active wetting: phase separation and binding maintained away from equilibrium. In synaptic vesicle clusters, synapsin 1, a membrane-associated protein, binds ATP and can undergo phase separation (42, 43). Stress granules are phase-separated condensates containing G3BP1 proteins that bind GTP and the lysosomal membrane (44, 45). Notably, stress granules can interact with lysosomes (8), making this system an appealing candidate for active wetting in a biological context.

The general prerequisite for active wetting and pronounced shape changes is that binding rate coefficients *k* are fast enough such that the reaction length scales $\lambda =\sqrt{D_{m}/k}$ are small and the binding flux localize well around the triple line. To be specific by the numbers, according to our model, pronounced shape changes occur for an activity parameter $|\chi_{\text{act}}|≃5$ (Fig. 7*D*), corresponding to a reaction–diffusion scale relative to droplet size $\lambda_{0}/X_{0}≃0.05$. This case could be realized, for example, by a surface diffusion constant, $D_{m}$, of 1μm^2^/s and a binding rate $k∼k_{0}e^{\chi_{\mathrm{act}}}$ in the order of 1/s. Thus, we propose a system that uses an ATP-driven phosphatase/kinase cycle to remove/donate a phosphate group to a phase-separating component and thereby controls binding (46). The turnover of ATP enables to actively regulate binding by changing the ATP concentration and thereby controlling the shape of wetted droplets experimentally. In a phase-separated system with active binding processes, a signature of active wetting that could be observed experimentally is a deviation of the droplet shape from a spherical cap. To this end, we suggest considering μm-sized droplets that are smaller than the capillary length such that gravitational effects on droplet shape are negligible. Moreover, concentration gradients in the vicinity of the triple line in experiments could also point to active wetting.

## Discussion

Various examples exist where wetting is affected through active processes. Dewetting of epithelial cells by their active propulsion (37) is one striking example. Another example is active droplets that adhere to surfaces and where chemical reactions are maintained away from equilibrium in the bulk (36). The common feature of these systems is that the active processes occur exclusively within the bulk (36, 37). This is fundamentally different from our work in which we propose a class of active systems where active binding processes affect wetting of droplets at the surface. We find that such binding processes give rise to flux patterns near the triple line at steady-state. While for a passive surface, the shape of wetted droplets is a spherical cap with a minimal surface area, flux loops adjacent to active surfaces deflect the triple line where all three phases coexist. This results in droplet shapes reminiscent of a pancake or a mushroom. A striking property is that the lower dimensional active surface can strongly affect the shape of the higher dimensional droplet.

We use a mapping of nonequilibrium fluids to electrostatic. Such a mapping has been used before to reveal simple principles governing nonequilibrium physics (47–49).

Our findings of shapes that significantly deviate from a spherical cap suggest that active wetting can deform and alter the structure of deformable membranes. We expect that such deformations can arise from the induced flux loops localized at the triple line acting as a local pump. Furthermore, such fluxes may drive membrane shape remodeling, including changes in membrane topology. Such changes would provide a gateway for biomolecular transport. Wetting on active surfaces and the associated transport phenomena thus may also have implications for a variety of cellular processes, including membrane budding (50), and vesicle rupture (51).

## Materials and Methods

We describe chemically active binding such that the binding flux becomes stronger or weaker than the passive system. In contrast, the unbinding flux remains unchanged, which leads to the following representation of the net desorption flux

[12] $$\begin{array}{cc} s & =k_{0}\left(1-\phi_{m}\right)\left(1-\phi_{0}\right)\\ & \;\times \left(\exp\left[\frac{\mu_{m}}{k_{B}T}\right]-\exp\left[\frac{\mu +\Delta \mu_{\mathrm{act}}}{k_{B}T}\right]\right), \end{array}$$

with *k*_0_ an intrinsic binding rate, *k* the Boltzmann constant and *T* the temperature. In an experimental setting, our model corresponds to a scenario where fuel, which drives the active binding process, partitions into the droplet and where fuel is continuously supplied from a reservoir while waste products are cleared sufficiently fast.

To study the wetting behavior, we describe both the bulk and the membrane by a Flory–Huggins free energy density, with

[13] $$\begin{array}{cc} f & =\frac{k_{B}T}{\nu }\left[\phi \ln\left(\phi \right)+\left(1-\phi \right)\ln\left(1-\phi \right)+\chi \phi \left(1-\phi \right)\right]\;, \end{array}$$

in bulk and

[14] $$\begin{array}{cc} f_{m} & =\frac{k_{B}T}{\nu_{m}}[\phi_{m}\ln\left(\phi_{m}\right)+\left(1-\phi_{m}\right)\ln\left(1-\phi_{m}\right)\\ & \;+\chi_{m}\phi_{m}\left(1-\phi_{m}\right)]\;, \end{array}$$

in the membrane and *χ*, $\chi_{m}$ the Flory–Huggins interaction parameters. The free energy *F* of the system, which is composed of the bulk with volume *V* and the membrane *m* now reads

[15] $$\begin{array}{cc} F[\phi ,\phi_{m}] & =\int_{V}d^{3}x\left[f\left(\phi \right)+\frac{\kappa }{2}{\left(\nabla \phi \right)}^{2}\right]\\ & \;+\int_{m}d^{2}x\left[f_{m}\left(\phi_{m}\right)+\frac{\kappa_{m}}{2}{\left(\nabla_{\|}\phi_{m}\right)}^{2}+\Omega \left(\phi_{0}\right)\right]\;, \end{array}$$

with *κ* and $\kappa_{m}$ accounting for the free energy cost of spatial inhomogeneities. The last term in Eq. **15** denotes the coupling energy between bulk and membrane. For simplicity, we restrict ourselves to a coupling that is linear in the bulk volume fraction at the surface, $\phi_{0}=\phi \left(z=0\right)$,

[16] $$\begin{array}{c} \Omega \left(\phi_{0}\right)=-\omega \phi_{0}\;. \end{array}$$

with a constant binding energy per unit area *ω*. The chemical potential in bulk and membrane are obtained from the free energy as μ/ν=δF/δϕ and $\mu_{m}/\nu_{m}=\delta F/\delta \phi_{m}$. We model the mobility coefficients as $\Lambda =\Lambda^{\left(0\right)}\phi \left(1-\phi \right)$ in bulk and $\Lambda_{m}=\Lambda_{m}^{\left(0\right)}\phi_{m}\left(1-\phi_{m}\right)$ on the membrane, with constant $\Lambda^{\left(0\right)}$, $\Lambda_{m}^{\left(0\right)}$. Minimizing *F* leads to the boundary condition Eq. **1C**, and with Eq. **16**, we have

[17] $$\begin{array}{c} n\cdot \nabla \phi {|}_{z=0}=-\frac{\omega }{\kappa }\;. \end{array}$$

To establish a relationship between the binding energy *ω* and the local contact angle *θ*_0_, we consider a sharp droplet interface, where the dense and dilute phases are homogeneous. The law of Young–Dupré then reads

[18] $$\begin{array}{c} \gamma_{0}\cos\theta_{0}=\omega \left(\phi^{I}-\phi^{\mathrm{II}}\right)\;. \end{array}$$

We define the reaction diffusion length scale $\lambda_{0}=\sqrt{\Lambda_{m}^{\left(0\right)}k_{B}T/k_{0}}$ as a characteristic length scale of the continuum model.

## Supplementary Material

Appendix 01 (PDF)

## Data Availability

All study data are included in the article and/or *SI Appendix*.

## References

[1] T. Young, An essay on the cohesion of fluids. Philos. Trans. R. Soc. **95**, 65–87 (1805).

[2] A. Dupré, P. Dupré, Théorie Mécanique de la Chaleur (Gauthier-Villars, 1869).

[3] P. G. de Gennes, Wetting: Statics and dynamics. Rev. Mod. Phys. **57**, 827–863 (1985).

[4] P. G. Gennes , Capillarity and Wetting Phenomena: Drops, Bubbles, Pearls, Waves (Springer, 2004).

[5] I. Dević, J. Encarnaci’on Escobar, D. Lohse, Equilibrium drop shapes on a tilted substrate with a chemical step. Langmuir **35**, 3880–3886 (2019). 30763107 10.1021/acs.langmuir.8b03557 PMC6427486

[6] G. Martouzet, L. Jørgensen, Y. Pelet, A. L. Biance, C. Barentin, Dynamic arrest during the spreading of a yield stress fluid drop. Phys. Rev. Fluids **6**, 044006 (2021).

[7] Y. Wu , Equilibrium droplet shapes on chemically patterned surfaces: Theoretical calculation, phase-field simulation, and experiments. J. Colloid Interface Sci. **606**, 1077–1086 (2022). 34487930 10.1016/j.jcis.2021.08.029

[8] Y. C. Liao , RNA granules hitchhike on lysosomes for long-distance transport, using annexin a11 as a molecular tether. Cell **179**, 147–164.e20 (2019). 31539493 10.1016/j.cell.2019.08.050 PMC6890474

[9] J. Agudo-Canalejo , Wetting regulates autophagy of phase-separated compartments and the cytosol. Nature **591**, 142–146 (2021). 33473217 10.1038/s41586-020-2992-3

[10] T. Lu , Endocytosis of coacervates into liposomes. J. Am. Chem. Soc. **144**, 13451–13455 (2022). 35878395 10.1021/jacs.2c04096 PMC9354246

[11] A. Mangiarotti, N. Chen, Z. Zhao, R. Lipowsky, R. Dimova, Wetting and complex remodeling of membranes by biomolecular condensates. Nat. Commun. **14**, 2809 (2023). 37217523 10.1038/s41467-023-37955-2 PMC10203268

[12] A. Mangiarotti , Biomolecular condensates modulate membrane lipid packing and hydration. Nat. Commun. **14**, 6081 (2023). 37770422 10.1038/s41467-023-41709-5 PMC10539446

[13] C. P. Brangwynne , Germline p granules are liquid droplets that localize by controlled dissolution/condensation. Science **324**, 1729–1732 (2009). 19460965 10.1126/science.1172046

[14] Y. Zhao, H. Zhang, Phase separation in membrane biology: The interplay between membrane-bound organelles and membraneless condensates. Dev. Cell **55**, 30–44 (2020). 32726575 10.1016/j.devcel.2020.06.033

[15] H. Kusumaatmaja, A. I. May, R. L. Knorr, Intracellular wetting mediates contacts between liquid compartments and membrane-bound organelles. J. Cell Biol. **220**, e202103175 (2021). 34427635 10.1083/jcb.202103175 PMC8404468

[16] O. Beutel, R. Maraspini, K. Pombo-Garcia, C. Martin-Lemaitre, A. Honigmann, Phase separation of zonula occludens proteins drives formation of tight junctions. Cell **179**, 923–936 (2019). 31675499 10.1016/j.cell.2019.10.011

[17] X. Zhao, G. Bartolucci, A. Honigmann, F. Jülicher, C. A. Weber, Thermodynamics of wetting, prewetting and surface phase transitions with surface binding. New J. Phys. **23**, 123003 (2021).

[18] K. Pombo-García, O. Adame-Arana, C. Martin-Lemaitre, F. Jülicher, A. Honigmann, Membrane prewetting by condensates promotes tight-junction belt formation. Nature **632**, 1–9 (2024). 10.1038/s41586-024-07726-0 PMC11324514 39112699

[19] D. Sun , Assembly of tight junction belts by ZO1 surface condensation and local actin polymerization. Dev. Cell. **60**, 1–17 (2025). 10.1016/j.devcel.2024.12.012 39742662

[20] X. Zhao, S. Liese, A. Honigmann, F. Jülicher, C. A. Weber, Theory of wetting dynamics with surface binding. New J. Phys. **26**, 103025 (2024).

[21] M. Moser, K. R. Legate, R. Zent, R. Fässler, The tail of integrins, talin, and kindlins. Science **324**, 895–899 (2009). 19443776 10.1126/science.1163865

[22] M. P. Christie, P. Simerska, F. E. C. Jen, M. P. Jennings, I. Toth, Liposomes for improved enzymatic glycosylation of lipid-modified lactose enkephalin. ChemPlusChem **78**, 793–796 (2013). 31986686 10.1002/cplu.201300115

[23] L. Harrington, J. M. Fletcher, T. Heermann, D. N. Woolfson, P. Schwille, De novo design of a reversible phosphorylation-dependent switch for membrane targeting. Nat. Commun. **12**, 1472 (2021). 33674566 10.1038/s41467-021-21622-5 PMC7935970

[24] J. D. Wurtz, C. F. Lee, Chemical-reaction-controlled phase separated drops: Formation, size selection, and coarsening. Phys. Rev. Lett. **120**, 078102 (2018). 29542937 10.1103/PhysRevLett.120.078102

[25] J. Berry, C. P. Brangwynne, M. Haataja, Physical principles of intracellular organization via active and passive phase transitions. Rep. Prog. Phys. **81**, 046601 (2018). 29313527 10.1088/1361-6633/aaa61e

[26] C. A. Weber, D. Zwicker, F. Jülicher, C. F. Lee, Physics of active emulsions. Rep. Prog. Phys. **82**, 064601 (2019). 30731446 10.1088/1361-6633/ab052b

[27] N. Ziethen, J. Kirschbaum, D. Zwicker, Nucleation of chemically active droplets. Phys. Rev. Lett. **130**, 248201 (2023). 37390433 10.1103/PhysRevLett.130.248201

[28] D. Zwicker, R. Seyboldt, C. A. Weber, A. A. Hyman, F. Jülicher, Growth and division of active droplets provides a model for protocells. Nat. Phys. **13**, 408–413 (2017).

[29] R. Seyboldt, F. Jülicher, Role of hydrodynamic flows in chemically driven droplet division. New J. Phys. **20**, 105010 (2018).

[30] J. Bauermann, C. A. Weber, F. Jülicher, Energy and matter supply for active droplets. Ann. Phys. **534**, 2200132 (2022).

[31] G. Bartolucci, O. Adame-Arana, X. Zhao, C. A. Weber, Econtrolling composition of coexisting phases via molecular transitions. Biophys. J. **120**, 4682–4697 (2021). 34600899 10.1016/j.bpj.2021.09.036 PMC8595902

[32] A. M. Bergmann , Liquid spherical shells are a non-equilibrium steady state. Nat. Commun. **14**, 6552 (2023). 37848445 10.1038/s41467-023-42344-w PMC10582082

[33] J. Bauermann, G. Bartolucci, J. Boekhoven, C. A. Weber, F. Jülicher, Formation of liquid shells in active droplet systems. Phys. Rev. Res. **5**, 043246 (2023).

[34] D. Zwicker, A. A. Hyman, F. Jülicher, Suppression of Ostwald ripening in active emulsions. Phys. Rev. E **92**, 012317 (2015). 10.1103/PhysRevE.92.012317 26274171

[35] J. Kirschbaum, D. Zwicker, Controlling biomolecular condensates via chemical reactions. J. R. Soc. Interface **18**, 20210255 (2021). 34186016 10.1098/rsif.2021.0255 PMC8241490

[36] N. Ziethen, D. Zwicker, Heterogeneous nucleation and growth of sessile chemically active droplets. J. Chem. Phys. **160**, 224901 (2024). 38856073 10.1063/5.0207761

[37] C. Pérez-González , Active wetting of epithelial tissues. Nat. Phys. **15**, 79–88 (2019). 31537984 10.1038/s41567-018-0279-5 PMC6753015

[38] J. Bauermann, S. Laha, P. M. McCall, F. Jülicher, C. A. Weber, Chemical kinetics and mass action in coexisting phases. J. Am. Chem. Soc. **144**, 19294–19304 (2022). 36241174 10.1021/jacs.2c06265 PMC9620980

[39] A. Bray, Theory of phase-ordering kinetics. Adv. Phys. **43**, 357–459 (1994).

[40] P. S. Schwarz , Parasitic behavior in competing chemically fueled reaction cycles. Chem. Sci. **12**, 7554–7560 (2021). 34163846 10.1039/d1sc01106e PMC8171353

[41] C. Donau, J. Boekhoven, The chemistry of chemically fueled droplets. Trends Chem. **5**, 45–60 (2023).

[42] M. Hosaka, T. C. Sïdhof, Synapsins I and II are ATP-binding proteins with differential Ca^2+^ regulation J. Biol. Chem. **273**, 1425–1429 (1998). 9430678 10.1074/jbc.273.3.1425

[43] D. Milovanovic, Y. Wu, X. Bian, P. D. Camilli, A liquid phase of synapsin and lipid vesicles. Science **361**, 604–607 (2018). 29976799 10.1126/science.aat5671 PMC6191856

[44] M. T. Prentzell , G3BPs tether the TSC complex to lysosomes and suppress mTORC1 signaling. Cell **184**, 655–674.e27 (2021). 33497611 10.1016/j.cell.2020.12.024 PMC7868890

[45] P. Yang , G3BP1 is a tunable switch that triggers phase separation to assemble stress granules. Cell **181**, 325–345.e28 (2020). 32302571 10.1016/j.cell.2020.03.046 PMC7448383

[46] L. B. Case, J. A. Ditlev, M. K. Rosen, Regulation of transmembrane signaling by phase separation. Annu. Rev. Biophys. **48**, 465–494 (2019). 30951647 10.1146/annurev-biophys-052118-115534 PMC6771929

[47] Y. Tsori, P. G. de Gennes, Self-trapping of a single bacterium in its own chemoattractant. Eur. Phys. Lett. **66**, 599 (2004).

[48] D. Deviri, S. A. Safran, Physical theory of biological noise buffering by multicomponent phase separation. Proc. Natl. Acad. Sci. U.S.A. **118**, e2100099118 (2021). 34135122 10.1073/pnas.2100099118 PMC8237649

[49] J. J. Christensen, K. Elder, H. C. Fogedby, Phase segregation dynamics of a chemically reactive binary mixture. Phys. Rev. E **54**, R2212–R2215 (1996). 10.1103/physreve.54.r2212 9965444

[50] R. Vutukuri , Active particles induce large shape deformations in giant lipid vesicles. Nature **586**, 52–56 (2020). 32999485 10.1038/s41586-020-2730-x

[51] S. Penič , Budding and fission of membrane vesicles: A mini review. Front. Phys. **8**, 342 (2020).

