# Supplementary material for: Chemically active wetting
Source: Proc Natl Acad Sci U S A. 2025 Apr 9;122(15):e2403083122. doi: 10.1073/pnas.2403083122 (PMC12012514; doi:10.1073/pnas.2403083122)
Supplement: Supplementary file 1 — Appendix 01 (PDF) [file pnas.2403083122.sapp.pdf]

Supplementary Information:
Chemically Active Wetting

Susanne Liese‡,[1] Xueping Zhao‡,[2] Christoph A. Weber*,[1] and Frank Jülicher*[3,4,5]

[1] Faculty of Mathematics, Natural Sciences, and Materials Engineering: Institute of Physics, University of Augsburg, Universitätsstraße 1, 86159 Augsburg, Germany
[2] Department of Mathematical Sciences, University of Nottingham Ningbo China, Taikang East Road 199, 315100 Ningbo, China
[3] Max Planck Institute for the Physics of Complex Systems, Nöthnitzer Straße 38, 01187 Dresden, Germany
[4] Center for Systems Biology Dresden, Pfotenhauerstraße 108, 01307 Dresden, Germany
[5] Cluster of Excellence Physics of Life, TU Dresden, 01062 Dresden, Germany

*To whom correspondence should be addressed. Email: christoph.weber@physik.uni-augsburg.de and julicher@pks.mpg.de

‡ These authors contributed equally to this work.

I. NON-DIMENSIONALIZATION

The governing equations of the system are given as:

$$\partial_t \phi_m = \nabla_{||} \cdot \left(D_m \, \phi_m (1-\phi_m) \nabla_{||} \frac{\mu_m}{k_{\mathrm{B}}T} \right) - s \,, \tag{S1a}$$

$$\partial_t \phi = \nabla \cdot \left(D \, \phi (1-\phi) \nabla \frac{\mu}{k_{\mathrm{B}}T} \right) \,, \tag{S1b}$$

where $(\ )_{||}$ depicts the 2D differential operator. The mobilities are modeled as $\Lambda_m = \Lambda_m^0 \phi_m(1-\phi_m)$ and $\Lambda = \Lambda^0 \phi(1-\phi)$ to ensure a diffusion equation with a constant diffusion coefficient in the dilute limit, and the respective solvent, where we define the diffusion coefficient as $D = k_{\mathrm{B}}T\Lambda^0$ and $D_{\mathrm{m}} = k_{\mathrm{B}}T\Lambda^0_{\mathrm{m}}$. The boundary conditions are given as

$$0 = \omega + \boldsymbol{n} \cdot \kappa \nabla \phi \,, \qquad x \in m \,, \tag{S1c}$$

$$0 = \boldsymbol{n} \cdot \kappa \nabla \phi \,, \qquad x \in \partial V \,, \tag{S1d}$$

$$0 = \boldsymbol{t} \cdot \kappa_m \nabla_{||} \phi_m \,, \qquad x \in \partial m \,, \tag{S1e}$$

$$-\frac{\nu}{\nu_m} s = \boldsymbol{n} \cdot \left(D\phi(1-\phi) \nabla \frac{\mu}{k_{\mathrm{B}}T} \right) \qquad x \in m \,, \tag{S1f}$$

$$0 = \boldsymbol{n} \cdot \left(D\phi(1-\phi) \nabla \frac{\mu}{k_{\mathrm{B}}T} \right) \,, \qquad x \in \partial V \,. \tag{S1g}$$

We set the characteristic length scale as $l_0 = \nu^{1/3}$, and time scale as $t_0 = \nu^{2/3}/D$. Using the rescaling $\tilde{\boldsymbol{x}} = \boldsymbol{x}/l_0$ and $\tilde{t} = t\, D/\nu^{2/3}$, our model has non-dimensional parameters:

$$\tilde{D}_{\mathrm{m}} = \frac{D_{\mathrm{m}}}{D} \,, \quad \tilde{k}_0 = k_0 \nu^{2/3}/D \,, \quad \tilde{\omega} = \frac{\omega}{k_{\mathrm{B}}T} \nu^{2/3} \,, \tag{S2}$$

$$\tilde{\kappa}_{\mathrm{m}} = \frac{1}{\nu^{2/3}} \kappa_{\mathrm{m}} \frac{\nu_{\mathrm{m}}}{k_{\mathrm{B}}T} \,, \quad \tilde{\kappa} = \kappa \frac{\nu^{1/3}}{k_{\mathrm{B}}T} \,. \tag{S3}$$

Furthermore, we introduce

$$\tilde{f} = \frac{\nu}{k_{\mathrm{B}}T} f \,, \quad \tilde{f}_{\mathrm{m}} = \frac{\nu_{\mathrm{m}}}{k_{\mathrm{B}}T} f_{\mathrm{m}} \,, \tag{S4}$$

the rescaled Flory-Huggings free energy density, with f, f_{m} given in Eqs. 11, 12 in the main text. And we set $\nu_{\mathrm{m}} = \nu^{2/3}$. For brevity, we skip the tildes in the following. The dimensionless equations governing of the kinetics of the system is given as:

$$\partial_t \phi_{\mathrm{m}} = \nabla_{\|} \cdot \left[D_{\mathrm{m}} \phi_{\mathrm{m}} (1-\phi_{\mathrm{m}}) \nabla_{\|} \left(\frac{\partial f_{\mathrm{m}}}{\partial \phi_{\mathrm{m}}} - \kappa_{\mathrm{m}} \nabla_{\|}^2 \phi_{\mathrm{m}} \right) \right] - k_0 (1-\phi_{\mathrm{m}})(1-\phi_0) \left[\exp\left[\frac{\partial f}{\partial \phi} - \kappa \nabla^2 \phi \right] - \exp\left[\frac{\partial f_{\mathrm{m}}}{\partial \phi_{\mathrm{m}}} - \kappa_{\mathrm{m}} \nabla_{\|}^2 \phi_{\mathrm{m}} + \chi_{\mathrm{act}} \phi_0 \right] \right] \,, \tag{S5a}$$

$$\partial_t \phi = \nabla \cdot \left[\phi (1-\phi) \nabla \left(\frac{\partial f}{\partial \phi} - \kappa \nabla^2 \phi \right) \right] \,, \tag{S5b}$$

**Parameter name**	**Symbol**	**rescaled value**
interaction coefficient in the membrane	χ_m	1
interaction coefficient in bulk	χ	2.5
binding energy per unit area	$\tilde{\omega}$	0.06
diffusion coefficient in the membrane	$\tilde{D}_{\rm m}$	1
gradient coefficient of molecule in the membrane	$\tilde{\kappa}_m$	1
gradient coefficient of molecule in the bulk	$\tilde{\kappa}$	1
binding rate	$\tilde{k}_0$	1
domain size of the bulk	$\tilde{L}\times\tilde{L}$	100×100

TABLE S1. **Model parameter and their dimensionless values in the model.**

with dimensionless boundary conditions:

$$0 = \omega + \boldsymbol{n}\cdot\kappa\nabla\phi\,, \qquad x\in m\,, \tag{S5c}$$

$$0 = \boldsymbol{n}\cdot\kappa\nabla\phi\,, \qquad x\in\partial V\,, \tag{S5d}$$

$$0 = \boldsymbol{t}\cdot\kappa_m\nabla_{\|}\phi_m\,, \qquad x\in\partial m\,, \tag{S5e}$$

$$-s = \boldsymbol{n}\cdot\left(\phi(1-\phi)\nabla\left[\frac{\partial f}{\partial\phi}-\kappa\nabla^2\phi\right]\right)\,, \qquad x\in m\,, \tag{S5f}$$

$$0 = \boldsymbol{n}\cdot\left(\phi(1-\phi)\nabla\left[\frac{\partial f}{\partial\phi}-\kappa\nabla^2\phi\right]\right)\,, \qquad x\in\partial V\,. \tag{S5g}$$

We list all the parameters and their dimensionless values in Table S1.

II. NUMERICAL SCHEME OF KINETIC MODEL

We solve the kinetic equations S5 with corresponding boundary conditions numerically. For the system S5 with the passive binding flux s, we initially employ the energy quadratization method [1, 2] to transform the system's free energy into a quadratic formula. Subsequently, we discretize the partial differential equations using a second-order finite difference method in space and the Crank-Nicolson method in time. A stabilizing term [3] is incorporated to facilitate larger time steps. Additionally, we apply the Euler method on the exponential terms in the active binding flux.

A. Numerical evaluation of the shape equation

To determine the drop shape based on the sharp interface model, we solve the shape equation, Eq. 5 in the main text. A schematic depiction of the arc length parameterization is shown in Fig. S1. As the expression for the chemical potential, Eq. 2 in the main text, diverges for $x\to X_{\rm p}$, $z\to 0$, we shift the start of the integration domain by a small displacement Δz, from the surface. To determine the shapes in shown in Fig. 2, we set $\Delta z = 0.001\ell$. The numerical simulations in Fig. 4 exhibit a finite droplet interface width. To optimally compare the droplet shape with the numerical simulations, we use the mesh size $\Delta\tilde{z} = 0.78$ in scaled units. This results in a shift of the starting point in the horizontal direction of $\Delta\tilde{x} = \Delta\tilde{z}/\tan(\theta_0)$ in scaled units.

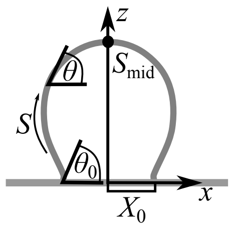

FIG. S1. Schematic depiction of the the arc length parameterization.

III. BOUNDARY CONDITIONS

A. Boundary condition of the flux

To derive the relation for the flux in bulk and the binding flux, we employ particle conservation. The total number of proteins in the system N reads:

$$N = \int_V d^3x \frac{\phi}{\nu} + \int_m d^2x \frac{\phi_\mathrm{m}}{\nu_\mathrm{m}}, \tag{S6}$$

with V the bulk volume and m the membrane area. Particle conservation implies $dN/dt = 0$, thus

$$\int_V d^3x \frac{\partial_t \phi}{\nu} = - \int_m d^2x \frac{\partial_t \phi_\mathrm{m}}{\nu_\mathrm{m}}. \tag{S7}$$

Inserting Eqs. 1 a,b) from the main text leads to

$$\frac{1}{\nu} \int_V d^3x \nabla \cdot \mathbf{j} = -\frac{1}{\nu_\mathrm{m}} \int_m d^2x \left[\nabla_\parallel \cdot \mathbf{j}_\mathrm{m} + s \right] \tag{S8}$$

Applying Gauss's theorem, we obtain:

$$\frac{1}{\nu} \int_{\partial V} d^2x \, \mathbf{n}_\mathrm{V} \cdot \mathbf{j} - \frac{1}{\nu} \int_m d^2x \, \mathbf{n} \cdot \mathbf{j} = -\frac{1}{\nu_\mathrm{m}} \int_{\partial m} dx \, \mathbf{t} \cdot \mathbf{j}_\mathrm{m} - \frac{1}{\nu_\mathrm{m}} \int_m d^2x \, s, \tag{S9}$$

with ∂V the surface of the volume V excluding the membrane, ∂m the perimeter of the membrane surface m, $\mathbf{n}_\mathrm{V}$ and $\mathbf{t}$ the outward pointing normal vectors on ∂V and ∂m, respectively and $\mathbf{n} = (0,0,1)^\mathrm{T}$ the normal vector of the membrane. In the far field gradients of the chemical potentials shall vanish. Eq. S9 simplifies to

$$\int_m d^2x \left[-\mathbf{n} \cdot \mathbf{j} + \frac{\nu}{\nu_\mathrm{m}} s \right] = 0. \tag{S10}$$

For the integral in Eq. S10 to vanish for an arbitrary membrane surface m the integrand has to vanish, which leads to the condition

$$\mathbf{n} \cdot \mathbf{j} = \frac{\nu}{\nu_\mathrm{m}} s. \tag{S11}$$

.

IV. ELECTROSTATIC ANALOGY

We consider a planar two-dimensional surface with a position dependent charge density $\rho(x,y)$. Above the surface is a charge free, linear dielectric medium. The medium below the surface is non-conducting and non-polarizable. According to Gauss's law the displacement field $\mathbf{D}$ and the charge density are related as

$$\nabla \cdot \mathbf{D} = \rho(x,y)\delta(z), \tag{S12}$$

where we place the charge layer at a height $z = 0$. To obtain the boundary condition at the surface, we now consider a small volume with height h and base area a. The volume is placed around the surface with the center of the volume at $z = 0$. Let the area a of the box be small enough so that the enclosed surface charge density can be taken as constant. Integration of Eq. S12 over the volume together with the Gaussian integral theorem and taking the limit $h \to 0$ leads to

$$a\mathbf{n} \cdot \mathbf{D} = \rho a, \tag{S13}$$

which is equivalent to

$$\mathbf{n} \cdot \mathbf{D} = \rho(x,y), \tag{S14}$$

V. DIPOLE POTENTIAL IN TWO AND THREE DIMENSIONS

To derive an analytic approximation for the chemical potential in bulk, we consider an electrostatics problem in two and three dimensions. In the following, we take the dielectric constant ϵ to be space independent.

A. Dipole Potential in Two Dimensions

To determine the electrostatic potential of a dipole in two dimensions, we start with a point charge that is places at lateral position X_p and height $z = 0$. The charge density ρ thus reads

$$\rho(x, z) = q\delta(x - X_\mathrm{p}, z). \tag{S15}$$

The electrostatic potential of the charge is obtained from

$$\Phi(x, z) = -\frac{1}{2\pi\epsilon}\int\int dx'dz'\rho(x', z')\ln\left(\sqrt{(x - x')^2 + (z - z')^2}\right), \tag{S16}$$

with leads directly to

$$\Phi(x, z) = -\frac{q}{2\pi\epsilon}\ln\left(\sqrt{(x - X_\mathrm{p})^2 + z^2}\right) \tag{S17}$$

Next, we construct a dipole with dipole moment $p_\mathrm{q} = qd$ from two point charges placed at distances $d/2$ around the lateral positions X_p. The charge density thus reads

$$\rho(x, z) = -q\delta\left(x - \left(X_\mathrm{p} - \frac{d}{2}\right), z\right) + q\delta\left(x - \left(X_\mathrm{p} + \frac{d}{2}\right), z\right). \tag{S18}$$

Taking the limit $d^2 \ll (x - X_\mathrm{p})^2 + z^2$ we find the dipole potential as

$$\Phi(x, z) = \frac{p_\mathrm{q}}{2\pi\epsilon}\frac{x - X_\mathrm{p}}{(x - X_\mathrm{p})^2 + z^2}. \tag{S19}$$

Using the mapping between electrostatics and wetting at active surfaces at steady state with $\Phi \to \mu_\mathrm{b}$, $p_\mathrm{q} \to p\frac{\nu}{\nu_\mathrm{m}}$ and $\epsilon \to \Lambda$, the chemical potential in bulk reads

$$\mu(x, z) = \frac{\bar{p}\nu}{2\pi\nu_\mathrm{m}\Lambda}\left[\frac{x - X_\mathrm{p}}{(x - X_\mathrm{p})^2 + z^2} - \frac{x + X_\mathrm{p}}{(x + X_\mathrm{p})^2 + z^2}\right]. \tag{S20}$$

B. Electrostatic Potential of a Dipole Ring

We consider the three dimensional case with a total charge qR_p that is homogeneously distributed along a circle with radius R_p at height $z = 0$. Expression the position vector $\mathbf{r}$ in cylindrical coordinates r, θ, z the charge line density $\rho(\mathbf{r}) = \frac{q}{2\pi}\delta(r - R_\mathrm{p}, z)$. The electrostatic potential of the charge distribution is obtained from

$$\Phi(\mathbf{r}) = \frac{1}{4\pi\epsilon}\int d^3r'\frac{\rho(\mathbf{r}')}{|\mathbf{r} - \mathbf{r}'|}. \tag{S21}$$

And the electrostatic potential reads

$$\Phi(r, \theta, z) = \frac{qR_\mathrm{p}}{8\pi^2\epsilon}\int_0^{2\pi} d\theta'\left[r^2 + R_\mathrm{p}^2 - 2rR_\mathrm{p}\cos(\theta - \theta') + z^2\right]^{-1/2} \tag{S22}$$

We rewrite the integrand using

$$r^2 + R_\mathrm{p}^2 - 2rR_\mathrm{p}\cos(\theta - \theta') = (r - R_\mathrm{p})^2 + 4rR_\mathrm{p}\sin^2\left(\frac{\theta - \theta'}{2}\right) \tag{S23}$$

and substitute the integration variable by $\tau = \frac{\theta - \theta'}{2}$. Since the system is rotationally symmetric the potential does not depend on θ and be can set without loss of generality $\theta = 0$ to obtain

$$\Phi(r, z) = \frac{qR_\mathrm{p}}{4\pi^2\epsilon\sqrt{(R_\mathrm{p} - r)^2 + z^2}}\int_0^{\pi} d\tau\left[1 + \frac{4rR_\mathrm{p}}{(R_\mathrm{p} - r)^2 + z^2}\sin^2\tau\right]^{-1/2}, \tag{S24}$$

which leads to

$$\Phi(r, z) = \frac{qR_\mathrm{p}}{2\pi^2\epsilon\sqrt{(R_\mathrm{p} - r)^2 + z^2}}K\left(-\frac{4rR_\mathrm{p}}{(R_\mathrm{p} - r)^2 + z^2}\right), \tag{S25}$$

with K the complete elliptic integral of the first kind.

Next, we consider two charged rings at distance d from each other. One ring with a charge $-qR_\mathrm{p}$ has radius of $R_\mathrm{p} - \frac{d}{2}$, while the second ring with charge qR_p has radius of $R_\mathrm{p} + \frac{d}{2}$. The charge density thus reads

$$\rho(\mathbf{r}) = \frac{-qR_\mathrm{p}}{2\pi(R_\mathrm{p} - d/2)}\delta(r - (R_\mathrm{p} - d/2), z) + \frac{qR_\mathrm{p}}{2\pi(R_\mathrm{p} + d/2)}\delta(r - (R_\mathrm{p} + d/2), z) \tag{S26}$$

and the electrostatic potential is directly obtained as

$$\Phi(r,z) = \frac{-qR_\mathrm{p}}{2\pi^2\epsilon\sqrt{\left(R_\mathrm{p}-\frac{d}{2}-r\right)^2+z^2}}K\left(-\frac{4r\left(R_\mathrm{p}-\frac{d}{2}\right)}{\left(R_\mathrm{p}-\frac{d}{2}-r\right)^2+z^2}\right) + \frac{qR_\mathrm{p}}{2\pi^2\epsilon\sqrt{\left(R_\mathrm{p}+\frac{d}{2}-r\right)^2+z^2}}K\left(-\frac{4r\left(R_\mathrm{p}+\frac{d}{2}\right)}{\left(R_\mathrm{p}+\frac{d}{2}-r\right)^2+z^2}\right). \tag{S27}$$

To obtain the potential of a dipole ring, we take the limit $d^2 \ll (r-R_\mathrm{p})^2+z^2$ and expand up to first order in d/R_p.

$$\Phi(r,z) = \frac{qdR_\mathrm{p}}{2\pi}\frac{1}{\pi\epsilon}\frac{(R_\mathrm{p}-r)R_\mathrm{p}}{((R_\mathrm{p}-r)^2+z^2)^{3/2}}\left[\frac{\pi rR_\mathrm{p}}{(R_\mathrm{p}-r)^2+z^2}{}_2F_1\left(\frac{3}{2},\frac{3}{2},2,-\frac{4rR_\mathrm{p}}{(R_\mathrm{p}-r)^2+z^2}\right)-K\left(-\frac{4rR_\mathrm{p}}{(R_\mathrm{p}-r)^2+z^2}\right)\right], \tag{S28}$$

with ${}_2F_1$ the hypergeometric function. Using the same analogy to electrostatics as in the two-dimensional case, we directly obtain the chemical potential in bulk as

$$\mu(r,z) = \frac{p_\mathrm{L}\nu}{\pi\nu_\mathrm{m}\Lambda}\frac{(R_\mathrm{p}-r)R_\mathrm{p}}{((R_\mathrm{p}-r)^2+z^2)^{3/2}}\left[\frac{\pi rR_0}{(R_\mathrm{p}-r)^2+z^2}{}_2F_1\left(\frac{3}{2},\frac{3}{2},2,-\frac{4rR_\mathrm{p}}{(R_\mathrm{p}-r)^2+z^2}\right)-K\left(-\frac{4rR_\mathrm{p}}{(R_\mathrm{p}-r)^2+z^2}\right)\right], \tag{S29}$$

where the line dipole moment is defined by

$$p_\mathrm{L} = \int_0^\infty dr\, r^2 s(r). \tag{S30}$$

C. Quadrupole moment

The quadrupole moment in cartesian coordinates in two and three dimensions reads

$$\text{2D: } Q_\mathrm{ij} = 2r_\mathrm{i}r_\mathrm{j} - r^2\delta_\mathrm{ij} \tag{S31a}$$

$$\text{3D: } Q_\mathrm{ij} = 3r_\mathrm{i}r_\mathrm{j} - r^2\delta_\mathrm{ij}. \tag{S31b}$$

For a charge distribution with $\rho(\mathbf{r}) = \rho(r)\delta(z-0)$, *i.e.* a symmetric charge distribution on a line for a two dimensional system and a rotationaly symmetric charge distribution on a plane for a three dimensional system the respective quadrupole moment reads

$$\text{2D: } \underline{\underline{Q}} = \begin{bmatrix} 2 & 0 \\ 0 & -2 \end{bmatrix}\int_0^\infty drr^2\rho(r), \tag{S32a}$$

$$\text{3D: } \underline{\underline{Q}} = \begin{bmatrix} \pi & 0 & 0 \\ 0 & \pi & 0 \\ 0 & 0 & -2\pi \end{bmatrix}\int_0^\infty drr^3\rho(r). \tag{S32b}$$

For the charge distributions discussed above, with

$$\text{2D: } \rho(r) = -q\delta\left(r-X_\mathrm{p}+\frac{d}{2}\right)+q\delta\left(r-X_\mathrm{p}-\frac{d}{2}\right), \tag{S33a}$$

$$\text{3D: } \rho(r) = \frac{-qR_\mathrm{p}}{2\pi(R_\mathrm{p}-d/2)}\delta\left(r-R_\mathrm{p}+\frac{d}{2}\right)+\frac{qR_\mathrm{p}}{2\pi(R_\mathrm{p}+d/2)}\delta\left(r-R_\mathrm{p}-\frac{d}{2}\right) \tag{S33b}$$

the quadrupole moment reads

$$\text{2D: } \underline{\underline{Q}} = \begin{bmatrix} 2 & 0 \\ 0 & -2 \end{bmatrix}2X_\mathrm{p}qd \tag{S34a}$$

$$\text{3D: } \underline{\underline{Q}} = \begin{bmatrix} \pi & 0 & 0 \\ 0 & \pi & 0 \\ 0 & 0 & -2\pi \end{bmatrix}\frac{2qdR_\mathrm{p}^2}{2\pi}. \tag{S34b}$$

Hence, to ensure that the local dipoles exhibit the same quadrupole moment as an arbitrary symmetric (2D), or rotationaly symmetric (3D) charge distribution $\rho(r)$, X_p and R_p are set by

$$\text{2D: } X_\mathrm{p} = \frac{\int_0^\infty dr\, r^2 \rho(r)}{2\int_0^\infty dr\, r\rho(r)}, \tag{S35a}$$

$$\text{3D: } R_\mathrm{p} = \frac{\int_0^\infty dr\, r^3 \rho(r)}{2\int_0^\infty dr\, r^2\rho(r)}. \tag{S35b}$$

VI. DIPOLE MOMENT p AND DIPOLE MOMENT POSITION X_p

In the following, we derive an expression for the dipole moment $\bar{p}$ and the dipole position X_p. To this end, it is helpful first to discuss three distinct lateral positions close to the triple point X_0, X_s and X_p, schematically depicted in Fig. S2. X_0 denotes the position of the droplet interface on the membrane, which we define as the position where the volume fraction has the value $\phi_{\frac{1}{2}} = \left(\phi^I + \phi^{II}\right)/2$. X_s denotes the position where the binding flux becomes zero. Furthermore, we define $\Delta X = X_\mathrm{s} - X_0$. The position of the dipole moment X_p is defined through the quadrupole moment, as discussed in the main text and further below. Since the magnitude of the binding flux is not symmetric around X_s, X_p is, in general, not equal to X_s.

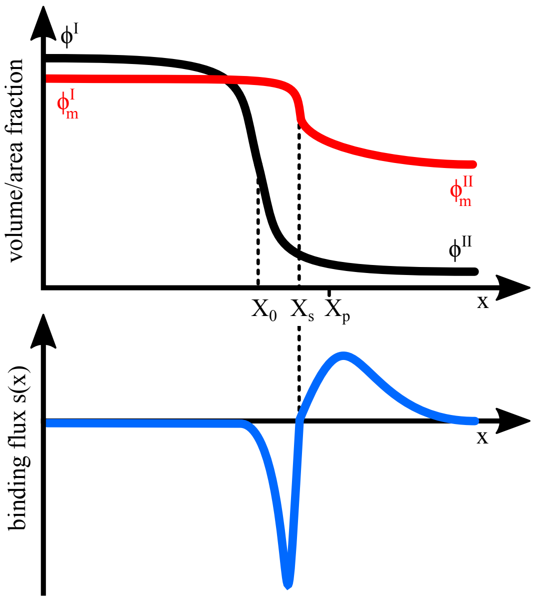

FIG. S2. Schematic depiction of the vicinity of the triple point: a) In the vicinity of the triple point, the bulk volume fraction at the membrane interface (black line) transitions from the equilibrium value in the dense phase ϕ^I to the equilibrium value in the dilute phase ϕ^{II}. The membrane area fraction (red line) transitions from ϕ_m^I to ϕ_m^{II}, where the values of ϕ_m^I, ϕ_m^{II} are set by the activity parameter χ_act. b) The binding flux s exhibits a maximum and a minimum in the vicinity of the triple point. The magnitude of s is, in general, not symmetric around X_s. The schematic depiction is not drawn to scale. The characteristic features correspond to a positive χ_act.

A. Shift between X_0 and X_s

First, we discuss the shift between the interface position in bulk, X_0, and in the membrane, X_s. We denote by ϕ_s the bulk volume fraction adjacent to X_s. For the binding flux to vanish the following condition has to be fulfilled

$$\mu_\mathrm{m}\big|_{X_\mathrm{s}} - \mu - \chi_\mathrm{act} k_\mathrm{B} T \phi_\mathrm{s} = 0. \tag{S36}$$

In the following, we assume that the bulk chemical potential μ can be considered constant and equal to the equilibrium value of the binodal phase separation. If gradients in the membrane area fraction are small, the membrane chemical potential is given by $\mu_\mathrm{m} = \nu_\mathrm{m} \frac{\partial f_\mathrm{m}}{\partial \phi_\mathrm{m}}$. Furthermore, we approximate the area fraction at X_s as $\phi_\mathrm{m}(X_\mathrm{s}) = (\phi_\mathrm{m}^I + \phi_\mathrm{m}^{II})/2$, which leads to

$$\phi_\mathrm{s} = \frac{\nu_\mathrm{m} \frac{\partial f_\mathrm{m}}{\partial \phi_\mathrm{m}}\Big|_{\frac{\phi_\mathrm{m}^I+\phi_\mathrm{m}^{II}}{2}} - \mu}{\chi_\mathrm{act} k_\mathrm{B} T} \tag{S37}$$

Next, we want to determine where the bulk volume fraction takes the value ϕ_s relative to the position of the interface X_0. To this end, we recapitulate an expression for the slope $\frac{d\phi}{dx}$ at the interface between a dense and a dilute phase in equilibrium. We consider the transition from a dense to a dilute phase across a planar interface. The surface free energy F reads

$$F = \int_{-\infty}^{\infty} dx \left[f(\phi) - f(\phi^{II}) - \frac{\mu}{\nu}\left(\phi - \phi^{II}\right) + \frac{\kappa}{2}\left(\frac{d\phi}{dx}\right)^2 \right] \tag{S38}$$

with x the coordinate perpendicular to the interface and where we place the interface at $x = 0$. In equilibrium the variation of F

$$\delta F = \int_{-\infty}^{\infty} dx \left[\frac{\partial f}{\partial \phi} - \frac{\mu}{\nu} - \kappa \frac{d^2\phi}{dx^2} \right] \delta\phi + \left[\kappa \frac{d\phi}{dx} \delta\phi \right]_{-\infty}^{\infty} \tag{S39}$$

has to vanish, which leads to

$$\frac{\partial f}{\partial \phi} - \frac{\mu}{\nu} - \kappa \frac{d^2\phi}{dx^2} = 0 \tag{S40}$$

or equivalently

$$\frac{d}{dx}\left(f - \frac{\mu}{\nu}\phi - \frac{\kappa}{2}\left(\frac{d\phi}{dx}\right)^2 \right) = 0. \tag{S41}$$

Since the slope $\frac{d\phi}{dx}$ has to vanish for $x \to \infty$, we find

$$\frac{d\phi}{dx} = -\sqrt{\frac{2}{\kappa}\left(f(\phi) - f(\phi^{II}) - \frac{\mu}{\nu}(\phi - \phi^{II}) \right)}. \tag{S42}$$

The slope at the interface X_0 thus reads

$$\left.\frac{d\phi}{dx}\right|_{X_0} = -\sqrt{\frac{2}{\kappa}\left(f(\phi_{\frac{1}{2}}) - f(\phi^{II}) - \frac{\mu}{\nu}(\phi_{\frac{1}{2}} - \phi^{II}) \right)}. \tag{S43}$$

As a last step, we assume that the slope $\frac{d\phi}{dx}$ in the interface region can be considered constant, which leads to the relation

$$\frac{\phi_\mathrm{s} - \phi_{\frac{1}{2}}}{X_\mathrm{s} - X_0} = \left.\frac{d\phi}{dx}\right|_{X_0}. \tag{S44}$$

The shift between X_0 and X_s, with $\Delta X = X_\mathrm{s} - X_0$ becomes

$$\Delta X = \frac{\frac{\phi^I + \phi^{II}}{2} - \left(\frac{\nu_\mathrm{m}}{k_\mathrm{B}T} \frac{\partial f_\mathrm{m}}{\partial \phi_\mathrm{m}}\Big|_{\frac{\phi_\mathrm{m}^I+\phi_\mathrm{m}^{II}}{2}} - \frac{\mu}{k_\mathrm{B}T} \right) / \chi_\mathrm{act}}{\sqrt{\frac{2}{\kappa}\left(f(\phi_{\frac{1}{2}}) - f(\phi^{II}) - \frac{\mu}{\nu}(\phi_{\frac{1}{2}} - \phi^{II}) \right)}}. \tag{S45}$$

B. Two-dimensional system

For the stationary solution, the binding flux s and the lateral membrane flux j_m are related as

$$s = -\frac{d}{dx} j_\mathrm{m}. \tag{S46}$$

Linearizing j_m around ϕ_m^I and ϕ_m^{II} respectively, leads to

$$j_\mathrm{m} = \begin{cases} -D_\mathrm{m}^I \frac{d}{dx}\phi_\mathrm{m}, & x < X_\mathrm{s} \\ -D_\mathrm{m}^{II} \frac{d}{dx}\phi_\mathrm{m}, & x > X_\mathrm{s}, \end{cases} \tag{S47}$$

with $D_\mathrm{m}^{I,II} = D_\mathrm{m}^{(0)} \phi_\mathrm{m}^{I,II}\left(1 - \phi_\mathrm{m}^{I,II}\right) \frac{\nu_\mathrm{m}}{k_\mathrm{B}T} \frac{\partial^2 f_\mathrm{m}}{\partial \phi_\mathrm{m}^2}\Big|_{\phi_\mathrm{m}^{I,II}}$ and $D_\mathrm{m}^{(0)} = \Lambda_\mathrm{m}^{(0)} k_\mathrm{B} T$. Linearising the binding flux s, we find

$$s = \begin{cases} k^I(\phi_\mathrm{m} - \phi_\mathrm{m}^I), & x < X_\mathrm{s} \\ k^{II}(\phi_\mathrm{m} - \phi_\mathrm{m}^{II}), & x > X_\mathrm{s}, \end{cases} \tag{S48}$$

with $k^{I,II} = k_0\left(1-\phi_{\mathrm{m}}^{I,II}\right)\left(1-\phi^{I,II}\right)\exp\left[\frac{\mu}{k_{\mathrm{B}}T}+\chi_{\mathrm{act}}\phi^{I,II}\right]\frac{\nu_{\mathrm{m}}}{k_{\mathrm{B}}T}\frac{\partial^2 f_{\mathrm{m}}}{\partial\phi_{\mathrm{m}}^2}\Big|_{\phi_{\mathrm{m}}^{I,II}}$, where we used a sharp interface model for the bulk with a homogeneous volume fraction ϕ^I inside and ϕ^{II} outside of the droplet. To solve Eq. S46 the membrane area fraction ϕ_{m} has to read

$$\phi_{\mathrm{m}} = \begin{cases}\phi_{\mathrm{m}}^I + C^I\exp\left[-\frac{x-X_{\mathrm{s}}}{\lambda^I}\right], & x < X_{\mathrm{s}}\\ \phi_{\mathrm{m}}^{II} + C^{II}\exp\left[-\frac{X_{\mathrm{s}}-x}{\lambda^{II}}\right], & x > X_{\mathrm{s}},\end{cases} \tag{S49}$$

with the reaction diffusion length scales $\lambda^{I,II} = \sqrt{D_{\mathrm{m}}^{I,II}/k^{I,II}}$ and the constants $C^{I,II}$. When written out, reaction diffusion lengths read

$$\lambda^{I,II} = \lambda_0\sqrt{\frac{\phi_{\mathrm{m}}^{I,II}}{\left(1-\phi^{I,II}\right)\exp\left[\frac{\mu}{k_{\mathrm{B}}T}+\chi_{\mathrm{act}}\phi^{I,II}\right]}}. \tag{S50}$$

Both the membrane area fraction and the membrane flux have to be continuous at X_{s}, which implies

$$\phi_{\mathrm{m}}^I + C^I = \phi_{\mathrm{m}}^{II} + C^{II} \tag{S51}$$

and

$$\frac{D_{\mathrm{m}}^I}{\lambda^I}C^I = -\frac{D_{\mathrm{m}}^{II}}{\lambda^{II}}C^{II}. \tag{S52}$$

From Eqs. S51 and S52 we find

$$C^I = -\left(\phi_{\mathrm{m}}^I - \phi_{\mathrm{m}}^{II}\right)\frac{D_{\mathrm{m}}^{II}\lambda^I}{D_{\mathrm{m}}^{II}\lambda^I + D_{\mathrm{m}}^I\lambda^{II}} \tag{S53a}$$

$$C^{II} = \left(\phi_{\mathrm{m}}^I - \phi_{\mathrm{m}}^{II}\right)\frac{D_{\mathrm{m}}^{I}\lambda^{II}}{D_{\mathrm{m}}^{II}\lambda^I + D_{\mathrm{m}}^I\lambda^{II}}. \tag{S53b}$$

Using Eqs. S48 and S49 the dipole moment is obtained as

$$\begin{aligned}\bar{p} &= \int_0^\infty dx\, xs(x)\\ &= k^I C^I\lambda^I\left(X_{\mathrm{s}}-\lambda^I\right) + k^{II}C^{II}\lambda^{II}\left(X_{\mathrm{s}}+\lambda^{II}\right)\\ &= -D_{\mathrm{m}}^I C^I + D_{\mathrm{m}}^{II}C^{II},\end{aligned} \tag{S54}$$

where we used Eq. S52 and the relation $k^{I,II}\lambda^{I,II} = D_{\mathrm{m}}^{I,II}/\lambda^{I,II}$. To simplify Eq. S54 further, we note that the area fraction at X_{s} is close to $(\phi_{\mathrm{m}}^I + \phi_{\mathrm{m}}^{II})/2$. The constants $C^{I,II}$ are thus approximated as

$$C^I = -\frac{\phi_{\mathrm{m}}^I - \phi_{\mathrm{m}}^{II}}{2} \tag{S55a}$$

$$C^{II} = \frac{\phi_{\mathrm{m}}^I - \phi_{\mathrm{m}}^{II}}{2}, \tag{S55b}$$

which simplifies the dipole moment to

$$\bar{p} = \left(D_{\mathrm{m}}^I + D_{\mathrm{m}}^{II}\right)\frac{\phi_{\mathrm{m}}^I - \phi_{\mathrm{m}}^{II}}{2}. \tag{S56}$$

Next, we determine the position of the dipole moment X_{p}, with

$$X_{\mathrm{p}} = \frac{\int_0^\infty dx\, x^2 s(x)}{2\int_0^\infty dx\, xs(x)}. \tag{S57}$$

Using Eqs. S48 and S49, we find

$$\int_0^\infty dx\, x^2 s(x) = -2\left(D_{\mathrm{m}}^I C^I\lambda^I + D_{\mathrm{m}}^{II}C^{II}\lambda^{II}\right) + X_{\mathrm{s}}\left(-D_{\mathrm{m}}^I C^I + D_{\mathrm{m}}^{II}C^{II}\right) - X_{\mathrm{s}}^2\left(\frac{D_{\mathrm{m}}^I}{\lambda^I}C^I + \frac{D_{\mathrm{m}}^{II}}{\lambda^{II}}C^{II}\right), \tag{S58}$$

where we again use $k^{I,II}\lambda^{I,II} = D_{\mathrm{m}}^{I,II}/\lambda^{I,II}$. The position of the dipole moment thus reads

$$X_{\mathrm{p}} = X_{\mathrm{s}} + \frac{D_{\mathrm{m}}^I C^I\lambda^I + D_{\mathrm{m}}^{II}C^{II}\lambda^{II}}{D_{\mathrm{m}}^I C^I - D_{\mathrm{m}}^{II}C^{II}}. \tag{S59}$$

Using Eq. S52, Eq. S59 simplifies to

$$X_{\mathrm{p}} = X_{\mathrm{s}} + \lambda^I - \lambda^{II}. \tag{S60}$$

The reaction diffusion length scales $\lambda^{I,II}$ depend on both $\phi_{\mathrm{m}}^{I,II}$ and $\phi^{I,II}$. Since $\phi_{\mathrm{m}}^{I,II}$ varies between 0 and 1 going from largely negative to largely positive χ_{act} the resulting $\lambda^{I,II}$ change significantly with χ_{act} as well (Fig. S3).

Together with Eq. S45 the final expression for the position of the dipole moment becomes

$$X_{\mathrm{p}} = X_0 + \Delta X + \lambda^I - \lambda^{II}. \tag{S61}$$

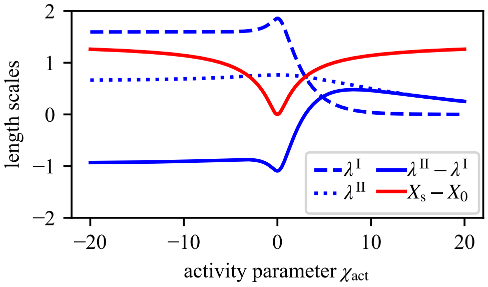

FIG. S3. Relevant length scales in connection to the dipole position: While the shift between the membrane interface and the droplet interface $X_s - X_0$ is symmetric with χ_{act}, neither λ^I nor λ^{II} exhibit such a symmetry. All lengths are given in units of the reaction diffusion length λ_0.

C. Three-dimensional system

For the stationary solution, the binding flux s and the radial component of the membrane flux $j_m^{(r)}$ are related as

$$s = -\frac{1}{r}\frac{d}{dr}\left(r\, j_m^{(r)}\right), \tag{S62}$$

with r the radial coordinate. Linearizing $j_m^{(r)}$ and s around ϕ_m^I and ϕ_m^{II} respectively, leads to

$$j_m = \begin{cases} -D_m^I \frac{d}{dr}\phi_m, & r < R_s \\ -D_m^{II} \frac{d}{dr}\phi_m, & r > R_s, \end{cases} \tag{S63}$$

and

$$s = \begin{cases} k^I(\phi_m - \phi_m^I), & r < R_s \\ k^{II}(\phi_m - \phi_m^{II}), & r > R_s, \end{cases} \quad , \tag{S64}$$

with R_s the radial position along the membrane, where the binding flux s is zero and $D_m^{I,II}$, $k^{I,II}$ the same diffusion constants and rates as in the two-dimensional case, Eqs. S47, S48. Eq. S62 is solved by

$$\phi_m = \begin{cases} \phi_m^I + \overline{C}^I \frac{I_0(r/\lambda^I)}{I_0(R_s/\lambda^I)}; & r < R_s, \\ \phi_m^{II} + \overline{C}^{II} \frac{K_0(r/\lambda^{II})}{K_0(R_s/\lambda^{II})} & r > R_s, \end{cases} \tag{S65}$$

with I_0, K_0 the modified Bessel function of first and second kind. The constants $\overline{C}^{I,II}$ are determined through the continuity of both the membrane area fraction and the membrane flux at R_s, which implies

$$\phi_m^I + \overline{C}^I = \phi_m^{II} + \overline{C}^{II} \tag{S66}$$

and

$$\frac{D_m^I}{\lambda^I}\overline{C}^I \frac{I_1(R_s/\lambda^I)}{I_0(R_s/\lambda^I)} = -\frac{D_m^{II}}{\lambda^{II}}\overline{C}^{II}\frac{K_1(R_s/\lambda^{II})}{K_0(R_s/\lambda^{II})}. \tag{S67}$$

From Eqs. S66 and S67 we find

$$\overline{C}^I = -\left(\phi_m^I - \phi_m^{II}\right)\frac{D_m^{II}\lambda^I I_0(R_s/\lambda^I)/I_1(R_s/\lambda^I)}{D_m^{II}\lambda^I I_0(R_s/\lambda^I)/I_1(R_s/\lambda^I) + D_m^I\lambda^{II}K_0(R_s/\lambda^{II})/K_1(R_s/\lambda^{II})} \tag{S68a}$$

$$\overline{C}^{II} = \left(\phi_m^I - \phi_m^{II}\right)\frac{D_m^I\lambda^{II}K_0(R_s/\lambda^{II})/K_1(R_s/\lambda^{II})}{D_m^{II}\lambda^I I_0(R_s/\lambda^I)/I_1(R_s/\lambda^I) + D_m^I\lambda^{II}K_0(R_s/\lambda^{II})/K_1(R_s/\lambda^{II})}. \tag{S68b}$$

We note that the following limits apply

$$\lim_{y\to\infty}\frac{I_0(y)}{I_1(y)} = 1 \tag{S69a}$$

$$\lim_{y\to\infty}\frac{K_0(y)}{K_1(y)} = 1. \tag{S69b}$$

For large droplets with $R_\mathrm{s}/\lambda^I \gg 1$, $R_\mathrm{s}/\lambda^{II} \gg 1$ the constants $\overline{C}^{I,II}$ in a three-dimensional system are equal to the constants $C^{I,II}$ in a two-dimensional system.
To determine the line dipole moment, it is useful to recapitulate two further mathematical relations involving the modified Bessel function of first and second kind:

$$\int_0^y dx\, x^2 I_0(x) = y^2 I_1(y) + \frac{\pi}{2} y I_1(y) \left[\frac{I_0(y)}{I_1(y)} L_1(y) - L_0(y)\right], \tag{S70a}$$

$$\int_y^\infty dx\, x^2 K_0(x) = \frac{\pi}{2} + y^2 K_1(y) - \frac{\pi}{2} y K_1(y) \left[\frac{K_0(y)}{K_1(y)} L_1(y) + L_0(y)\right], \tag{S70b}$$

with $L_\alpha(y)$ the modified Struve function. Using the limits

$$\lim_{y\to\infty} \frac{I_0(y)}{I_1(y)} L_1(y) - L_0(y) = -\frac{2}{\pi}, \tag{S71a}$$

$$\lim_{y\to\infty} \frac{1}{yK_1(y)} - \frac{K_0(y)}{K_1(y)} L_1(y) - L_0(y) = \frac{2}{\pi}, \tag{S71b}$$

we obtain the asymptotic behavior

$$\int_0^y dx\, x^2 I_0(x) \approx (y^2 - y) I_1(y), \text{ for } y \gg 1, \tag{S72a}$$

$$\int_y^\infty dx\, x^2 K_0(x) \approx (y^2 + y) K_1(y), \text{ for } y \gg 1. \tag{S72b}$$

Using Eqs. S64, S65 and S72 the line dipole moment for large droplets ($R_\mathrm{s}/\lambda^I \gg 1$, $R_\mathrm{s}/\lambda^{II} \gg 1$) is obtained as

$$\begin{aligned}
\bar{p}_L &= \int_0^\infty dr\, r^2 s(r) \\
&= k^I \overline{C}^I \frac{I_1\left(R_\mathrm{s}/\lambda^I\right)}{I_0\left(R_\mathrm{s}/\lambda^I\right)} \left(R_\mathrm{s}^2 \lambda^I - R_\mathrm{s}\left(\lambda^I\right)^2\right) + k^{II} \overline{C}^{II} \frac{K_1\left(R_\mathrm{s}/\lambda^{II}\right)}{K_0\left(R_\mathrm{s}/\lambda^{II}\right)} \left(R_\mathrm{s}^2 \lambda^{II} - R_\mathrm{s}\left(\lambda^{II}\right)^2\right) \\
&= \left(D_\mathrm{m}^{II} \overline{C}^{II} \frac{K_1\left(R_\mathrm{s}/\lambda^{II}\right)}{K_0\left(R_\mathrm{s}/\lambda^{II}\right)} - D_\mathrm{m}^I \overline{C}^I \frac{I_1\left(R_\mathrm{s}/\lambda^I\right)}{I_0\left(R_\mathrm{s}/\lambda^I\right)}\right) R_\mathrm{s}
\end{aligned} \tag{S73}$$

where we used Eq. S67 and the relation $k^{I,II}\lambda^{I,II} = D_\mathrm{m}^{I,II}/\lambda^{I,II}$.
Using Eq. S69, Eq. S73 is further simplified

$$\bar{p}_L = \left(D_\mathrm{m}^{II} C^{II} - D_\mathrm{m}^I C^I\right) R_\mathrm{s}, \tag{S74}$$

with $C^{I,II}$ given in Eq. S53. Comparing the dipole moment $\bar{p}$ in a two-dimensional system, Eq. S54 and the line dipole moment $\bar{p}_\mathrm{L}$ in a three-dimensional system, we find

$$\bar{p}_\mathrm{L} = R_\mathrm{s} \bar{p}. \tag{S75}$$

Next, we determine the position of the dipole moment R_p, with

$$R_\mathrm{p} = \frac{\int_0^\infty dr\, r^3 s(r)}{2\int_0^\infty dr\, r^2 s(r)}. \tag{S76}$$

We use the relations

$$\int_0^y dx\, x^3 I_0(x) = \left(y^3 + 4y\right) I_1(y) - 2y^2 I_0(y), \tag{S77a}$$

$$\int_y^\infty dx\, x^3 K_0(x) = \left(y^3 + 4y\right) K_1(y) + 2y^2 K_0(y), \tag{S77b}$$

to evaluate the integral

$$\begin{aligned}
\int_0^\infty dr\, r^3 s(r) &= k^I \overline{C}^I \left[\left(R_\mathrm{s}^3 \lambda^I + 4R_\mathrm{s}(\lambda^I)^3\right) \frac{I_1(R_\mathrm{s}/\lambda^I)}{I_0(R_\mathrm{s}/\lambda^I)} - 2R_\mathrm{s}^2(\lambda^I)^2\right] + k^{II} \overline{C}^{II} \left[\left(R_\mathrm{s}^3 \lambda^{II} + 4R_\mathrm{s}(\lambda^{II})^3\right) \frac{K_1(R_\mathrm{s}/\lambda^{II})}{K_0(R_\mathrm{s}/\lambda^{II})} + 2R_\mathrm{s}^2(\lambda^{II})^2\right] \\
&= 4R_\mathrm{s}\left(D_\mathrm{m}^I \lambda^I \overline{C}^I \frac{I_1(R_\mathrm{s}/\lambda^I)}{I_0(R_\mathrm{s}/\lambda^I)} + D_\mathrm{m}^{II} \lambda^{II} \overline{C}^{II} \frac{K_1(R_\mathrm{s}/\lambda^{II})}{K_0(R_\mathrm{s}/\lambda^{II})}\right) + 2R_\mathrm{s}^2 \left(D_\mathrm{m}^{II} \overline{C}^{II} - D_\mathrm{m}^I \overline{C}^I\right),
\end{aligned} \tag{S78}$$

where we used Eq. S67 and the relation $k^{I,II}\lambda^{I,II} = D_\mathrm{m}^{I,II}/\lambda^{I,II}$. For large droplets, with $R_\mathrm{s}/\lambda^I, R_\mathrm{s}/\lambda^{II} \gg 1$ Eq. S78 simplifies to

$$\int_0^\infty dr\, r^3 s(r) = 4R_\mathrm{s}\left(D_\mathrm{m}^I \lambda^I C^I + D_\mathrm{m}^{II} \lambda^{II} C^{II}\right) + 2\left(D_\mathrm{m}^{II} C^{II} - D_\mathrm{m}^I C^I\right) R_\mathrm{s}^2. \tag{S79}$$

Thus the position of the dipole moment reads

$$R_{\mathrm{p}} = R_{\mathrm{s}} + 2\left(\lambda^{I} - \lambda^{II}\right). \tag{S80}$$

Together with Eq. S45 the final expression for the position of the dipole moment becomes

$$R_{\mathrm{p}} = R_0 + \frac{\frac{\phi^{I}+\phi^{II}}{2} - \left(\frac{\nu_{\mathrm{m}}}{k_{\mathrm{B}}T}\frac{\partial f_{\mathrm{m}}}{\partial \phi_{\mathrm{m}}}\Big|_{\frac{\phi_{\mathrm{m}}^{I}+\phi_{\mathrm{m}}^{II}}{2}} - \frac{\mu}{k_{\mathrm{B}}T}\right)/\chi_{\mathrm{act}}}{\sqrt{\frac{2}{\kappa}\left(f(\phi_{\frac{1}{2}}) - f(\phi^{II}) - \frac{\mu}{\nu}(\phi_{\frac{1}{2}} - \phi^{II})\right)}} + 2\left(\lambda^{I} - \lambda^{II}\right), \tag{S81}$$

with R_0 the base radius of the droplet.

---

[1] X. Zhao and Q. Wang, A second order fully-discrete linear energy stable scheme for a binary compressible viscous fluid model, Journal of Computational Physics **395**, 382 (2019).

[2] J. Zhao, X. Yang, Y. Gong, X. Zhao, X. Yang, J. Li, and Q. Wang, A general strategy for numerical approximations of non-equilibrium models-part i: Thermodynamical systems, International Journal of Numerical Analysis & Modeling **15**, 884 (2018).

[3] J. Shen and X. Yang, Numerical approximations of allen-cahn and cahn-hilliard equations, Discrete and Continuous Dynamical Systems **28**, 1669 (2010).
